# A general model for predicting the binding affinity of reversibly and irreversibly dimerized ligands

**DOI:** 10.1371/journal.pone.0188134

**Published:** 2017-11-22

**Authors:** Kenneth W. Foreman

**Affiliations:** BlinkBio, Inc., Jupiter, FL, United States of America; University of the Basque Country, SPAIN

## Abstract

Empirical data has shown that bivalent inhibitors can bind a given target protein significantly better than their monomeric counterparts. However, predicting the corresponding theoretical fold improvements has been challenging. The current work builds off the reacted-site probability approach to provide a straightforward baseline reference model for predicting fold-improvements in effective affinity of dimerized ligands over their monomeric counterparts. For the more familiar irreversibly linked bivalents, the model predicts a weak dependence on tether length and a scaling of the effective affinity with the 3/2 power of the monomer’s affinity. For the previously untreated case of the emerging technology of reversibly linking dimers, the effective affinity is also significantly improved over the affinity of the non-dimerizing monomers. The model is related back to experimental quantities, such as EC_50_s, and the approaches to fully characterize the system given the assumptions of the model. Because of the predicted significant potency gains, both irreversibly and reversibly linked bivalent ligands offer the potential to be a disruptive technology in pharmaceutical research.

## Introduction

The basis for expecting success in targeted pharmacological therapies has implicitly rested on the assumption of the existence of a relatively small, well-defined pocket to which a molecule with “drug-like” properties can bind. These properties have been statistically analyzed to determine which ones differentiate drugs from mere chemicals, the most familiar of which is the Rule-of-5 (RO5) [1]. A molecular weight cut-off at 500 Daltons in the RO5, coupled with the maximum binding energy gain expected per atom [2–4], implies one can determine how “druggable” any particular stretch of protein surface is [5, 6]. For certain surfaces, such as protein-protein interfaces, the predicted druggability is low due to the improbability of finding a low molecular weight binder of sufficient efficacy [7]. In order to overcome this drawback and achieve the necessary potencies and selectivities for advancing research against traditionally more difficult targets, many researchers have begun employing bivalents, molecules with a typically flexible tether or connector that joins two ligands, to simultaneously bind distinct pockets on one or more target molecules [8–11].

Since bivalents have two independent binding elements that are now correlated through distance constraints, their behavior in assays may diverge, even significantly, from those of mixtures of the ligands themselves. Indeed, dramatic improvements in potency against various biological targets have been observed [8, 11–14]. Several theoretical models have been formulated which describe the effects of binding to irreversibly connected bivalents [15–22], although mostly in the context of polyvalent antibody interactions. The easiest model to understand follows the stepwise addition approach [19], which describes the thermodynamics of the formation of higher order complexes through the thermodynamics of single ligand addition to lower order complexes. Although very straightforward to describe, an infinite number of complexes are possible for bivalent ligands interacting with bivalent targets, thus complicating the mathematics involved in detailing the system’s equilibria. An alternative to stepwise addition is the reacted-site probability approach [18], which describes the various equilibria as a function of the probability of any particular target site being occupied by a ligand. Although the two approaches yield essentially identical results [18], the reacted-site probability method is easier to work with mathematically, but perhaps harder to conceptualize, particularly for polyvalent ligands interacting with polyvalent targets. Both approaches have focused on non-cyclic structures whenever the valence is at least two for both ligand and target. Additionally, earlier efforts focused on determining critical concentrations at which the formation of higher order structures dominates over the formation of complexes in which the bivalent straddles both sites of the same target molecule. Using either approach to find the predicted fold improvement due to avidity is a challenge in most cases.

Predicting the affinity increases of self-assembling bivalents is becoming more relevant, as click chemistries [23] start with monomers and attempt to create irreversibly connected bivalents on the target. Clearly, this approach takes advantage of the relatively fast on-rates of monomers and of the relatively slow off-rates of bivalents. Nevertheless, the irreversible formation of dimer dictates that their thermodynamic treatment involves only irreversibly linked bivalents as described above. Reversible bioorthogonal moieties were reviewed in the literature [24, 25] and additional ones have been introduced by Barany et al. [26, 27]. Recent work shows that bivalents employing these reversible moieties in the linkers as part of a reversible linker technology can also penetrate cells and dimerize to yield significant activity gains [27]. These reversible linkers require a more complex equilibrium description since several of these chemistries are completely reversible under aqueous conditions. None of the earlier thermodynamic treatments handles such bivalents with dissociable tethers. Hence models which anticipate reversibly linked monomer-dimer equilibrium behavior, predict fold-improvement of the effective binding affinity upon dimerization, and compare straightforwardly against equivalent irreversibly linked bivalents are needed.

In this paper, we describe the predicted avidity effects of reversible covalent interactions between two identical ligands simultaneously bound to targets such as proteins. We build on the reacted-site probability approach to describe the scenario in which two identical non-interacting ligands bind two separate sites while including a distance-dependent model for both irreversibly and reversibly dimerized monomers. The model is related back to experimentally determinable binding affinities. We find that introducing bivalency increases effective affinity to targets with two equivalent binding sites by a factor of ten for every halving of the connector length or for every 100-fold increase in monomer affinity for the binding site. For reversibly forming dimers, we find that meaningful affinity increases are also predicted, regardless of ligand affinity, for connectors below a certain length, suggesting the potential high utility of both irreversibly and reversibly tethered monomers, especially against traditionally “undruggable” targets.

## Reacted site method definitions and terminology

The reacted-site probability approach [18] is reviewed in more detail below, as it provides useful insights in the more complex scenarios. The familiar case of a target with a single binding site combining with a monovalent ligand provides a basis for treating more complex systems.

### Single binding site, monovalent binder

In the reacted-site probability approach, the calculation of thermodynamic quantities, such as the association constant K_A_ (or dissociation constant K_D_ = 1/K_A_) between a target T and a ligand L depends on the probabilities F_T_ that a target binding site is occupied and F_L_ that a ligand moiety is bound to the target. F_T_ is defined as the ratio of target sites bound to total target sites available at equilibrium; a similar definition, involving ligands, holds for F_L_. Given a starting ligand concentration L_0_, mixed with a target at starting concentration T_0_, the governing equations are, for mass balance,

$$T_{0}=T_{f}+F_{T}T_{0}$$ (1)

$$L_{0}=L_{f}+F_{L}L_{0}$$ (2)

and for “site occupancy balance”,

$$F_{T}T_{0}=F_{L}L_{0}$$ (3)

where T_f_ and L_f_ are the unbound target and ligand concentrations, respectively, after equilibrating the combined components. For thermodynamic equilibrium,

$$K_{A}=\frac{F_{T}T_{0}}{\left(T_{f}\right)\left(L_{f}\right)}=\frac{F_{T}T_{0}}{\left[T_{0}\left(1-F_{T}\right)\right]\left[L_{0}\left(1-F_{L}\right)\right]}=\frac{F_{T}}{\left(1-F_{T}\right)\left(L_{0}-F_{T}T_{0}\right)},$$ (4)

which permits the calculation of the probability of the target site being occupied by L, given L_0_, T_0_ and K_A_. Typically, the association constant is unknown and needs to be determined, either through direct or indirect experimental measures of the fraction R of the total target sites occupied by the ligand given the starting concentrations of target and ligand. Finding R permits theory to be connected to experiment via

$$R=\frac{F_{T}T_{0}}{T_{0}}=F_{T}.$$ (5)

When R = ½, Eq 4 simplifies to

$$K_{A}=\frac{1}{\left(L_{0}-T_{0}/2\right)}$$ (6)

which, when rearranged, produces the more familiar form

$$L_{0}-T_{0}/2=K_{D},$$ (7)

where L_0_ is the concentration which yields the experimentally determined 50% binding at a given T_0_. We refer to this L_0_ as the EC_50_ at T_0_, or just EC_50_ for short, but it may be referred to as an IC_50_ when employing competition assay formats.

### Multiple binding sites, monovalent binder

When L can bind to more than one available site on T, the descriptions become more complex. The probability of binding any site on T becomes a function of the affinity of L for each site on an uncomplexed target, as well as a dependent probability for the binding of L to a partially complexed target. The most idealized scenario assumes that all sites on the target are equivalent and independent, meaning that affinity of L towards each site is the same and does not change with the binding of L to any of the other sites, and that the sites are always available to interact with L, independent of the occupancy of other sites with other copies of L. This scenario is most closely approached when a protein contains multiple copies of a particular domain or when homo-oligomerization occurs. For example, tryptase forms a dimer of dimeric functional units, each of which is catalytically competent, independent of the occupancy of the other dimeric unit [28]. Similarly, BRD4 contains a tandem repeat of its bromodomain, each of which can equally well bind acetylated lysine independent of the occupancy of the other [29].

In the reacted-site probability approach, the fraction of ligand bound remains the same conceptually as in the single binding site model, but now the fractions of singly (F_T1_) and doubly (F_T2_) occupied target needs to be considered. Since the sites are independent, the fractions are simply the products of the fractions for the states of the two sites, scaled by the number of ways such states may be achieved. Particularly,

$$F_{T1}=2F_{T}\left(1-F_{T}\right)\;\mathrm{and}$$ (8)

$$F_{T2}=F_{T}^{2}.$$ (9)

The stoichiometric mass balance equation becomes

$$T_{0}\left(F_{T1}+2F_{T2}\right)=F_{L}L_{0},$$ (10)

which, when substituted with Eqs 8 and 9, reduces to the expected

$$2F_{T}T_{0}=F_{L}L_{0}.$$ (11)

Taking any definition of K_D_ for a single ligand coming off and letting T_1_ be the concentration of target with one ligand bound to the first site on the target, one obtains, for example,

$$K_{D}=\frac{T_{f}L_{f}}{T_{1}}=\frac{T_{0}{\left(1-F_{T}\right)}^{2}L_{0}\left(1-F_{L}\right)}{T_{0}F_{T1}/2}=\frac{\left(1-F_{T}\right)L_{0}\left(1-2F_{T}T_{0}/L_{0}\right)}{F_{T}},$$ (12)

where mass balance equations and substitutions from Eqs 8, 9 and 11 were employed to obtain 12. The left hand side of Eq 11 defines the total number of target sites bound, while 2T_0_ is the total number of sites possible. Thus Eq 5 still holds for all targets with two binding sites, and, in fact, can be shown to hold no matter how many sites exist on a given target, provided the assumption of independence is retained. Hence, when R = ½, Eq 12 reduces to

$$L_{0}-T_{0}=K_{D}$$ (13)

Since the single and dual binding site models feature the same K_D_, the relationship between the EC_50,mm_ from the single binding site model (Eq 7) and the EC_50,bm_ from the dual binding site model (Eq 13) is

$$EC_{50,bm}=EC_{50,mm}+T_{0}/2.$$ (14)

Typically, the target concentration is much less than the EC_50_ and both model systems produce essentially identical results, as expected. Alternately, identical numerical EC_50_’s can be obtained by assaying the two binding site system at half the target concentration of that in the one binding site system.

### Single binding site, bivalent binder

Much like when a target contains multiple sites, when a ligand molecule is multivalent, it may be treated as multiple ligands connected by tethers. The probability of binding a target site becomes a dependent function of how many ligands are bound, which ligands are bound, and the steric constraints of that arrangement. The strongest simplifying assumptions one can make are that the tethers connecting any two ligands do not alter the affinity of the ligand for its site; that interactions of the tether with the target do not contribute to the affinity of the ligand for the target; and that the probability of a ligand binding a site remains constant independent of what other ligands are bound. For ligands that are known to bind independently when co-dosed as separate ligands, and when their tethers are “out to solvent”, these simplifications are well justified and are likely satisfied in designs for, e.g., cIAP bivalent inhibitors for which it was suggested that only one binding site is involved in the binding of these bivalent inhibitors[9].

Swapping every T and L for the other in Eqs 8–12 provides the equations for the reacted-site probability approach for this model. Algebraic manipulation leads to

$$2L_{0}-T_{0}/2=K_{D},$$ (15)

where the multiplicative factor of 2 comes from the number of ligands present on each molecule. The simplest extrapolation, from Eqs 13 and 15, for a system containing multivalent molecules with p ligands and targets with s sites, would be the relation $pL_{0}-sT_{0}/2=K_{D}$. As will be demonstrated, this relation only appears to hold when ligand non-interaction is assumed. Since the single and dual binding site models feature the same K_D_, the relationship between the EC_50,mm_ from the single binding site model (Eq 7) and the EC_50,mb_ from the dual binding site model (Eq 15) is

$$EC_{50,mb}=EC_{50,mm}/2.$$ (16)

The factor of two difference in Eq 16 is readily understood based on the fact that two ligands are present in the bivalent model, while only one is present in the monovalent model. Within the context of these models, this difference means multivalents with p ligands will have EC_50_s p times more potent against single site targets than their monovalent ligand counterparts. Thus, if the approximations used in the above derivations can be realized (e.g., minimizing the interference from the tether), then multivalents which can attain other favorable properties such as cellular uptake, and possibly oral bioavailability, have great therapeutic potential.

## Results and discussion

### Dual binding sites, bivalent ligand

The reacted-site probability approach ignores the potential influence of tethers on the probability of monomers from the same bivalent ligand binding to the same target when it contains dual binding sites. A model which takes this influence into account explicitly is expanded below.

**Fig 1:**
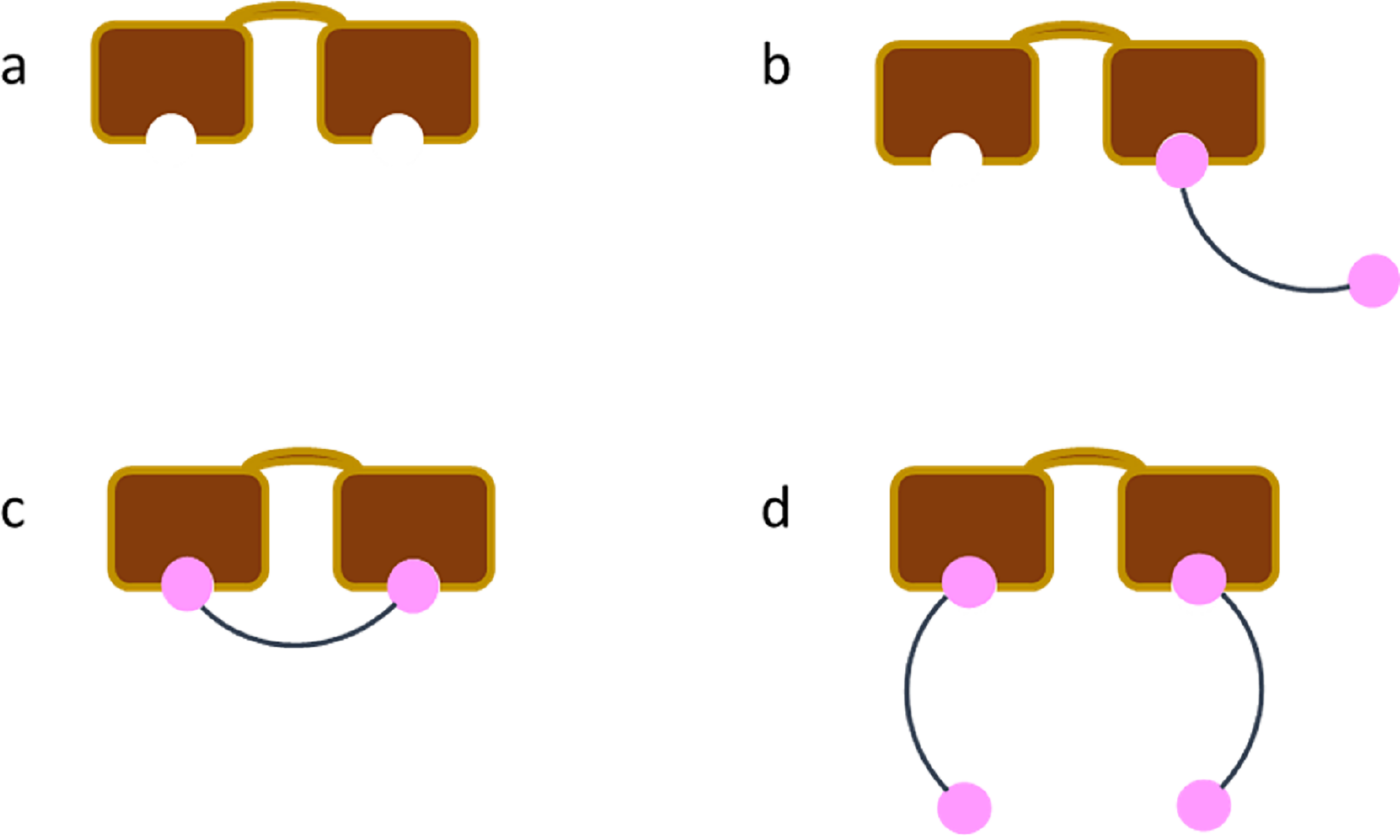
Schematic representation of the possible interactions between a single two site target and bivalent molecules. The target (brown) may have no ligand (pink) bound (a), only one ligand bound [only one of the two possible arrangements is depicted] (b), or two ligands bound. For the later scenario, the two bound ligands could be from the same bivalent molecule (c) or from two separate bivalent molecules (d). Whenever a ligand is depicted as unbound, it is done so as a convenience, since its binding state is irrelevant to determining the probability of site occupancy on an individual target molecule.

Let F_2u_ be the fraction of completely unbound target (Fig 1*A*), F_1u_ the fraction of singly bound target (Fig 1*B*), F_22_ the fraction of target with two bivalents bound (Fig 1*D*), and F_21_ the fraction of target with one bivalent occupying both sites (Fig 1*C*, the 1:1 state). Clearly their sum must equal 1. Of the fraction which is not in the 1:1 state, the distribution must behave as in the single ligand, two site target model, namely

$$F_{2u}={\left(1-F_{T}\right)}^{2}\left(1-F_{21}\right),$$ (17)

$$F_{1u}=2F_{T}\left(1-F_{T}\right)\left(1-F_{21}\right),$$ (18)

$$F_{22}=F_{T}^{2}\left(1-F_{21}\right),$$ (19)

where F_T_ is the fraction of targets sites not occupied by the 1:1 state but otherwise occupied by bivalent. Based on this definition then, the total fraction of occupied sites must be

$$R=F_{21}+\left(1-F_{21}\right)F_{T},$$ (20)

which implies F_T_ may be related to F_21_ via

$$F_{T}=\frac{R-F_{21}}{1-F_{21}}.$$ (21)

In order to introduce the tether length dependence and resolve the value for F_21_, we utilized a two-step thermodynamic process. In the first step, we permitted all processes of binding between the bivalent and the target, except the 1:1 state. After equilibrating the system, the second step permits formation of the 1:1 state. The final fraction of singly bound bivalent to singly bound target (Fig 1*D*) after the second step must equal the starting concentration of singly bound bivalent to singly bound target after the first step, multiplied by the fraction that does not form a 1:1 complex in the second step. The fraction F_1u_ in Eq 18 lacks a specification of the state of the bivalent bound. Specifically, the other ligand may be unbound, as illustrated in Fig 1B, or may be bound to a separate target molecule. This latter scenario cannot lead to the 1:1 state without first having the separate target molecule dissociate. Therefore, only a subset of the T_0_F_1u_ molecules has ligands bound to only the one target. Much as in the single binding site, bivalent binder description, one may write equivalent expressions for the ligand occupancies by replacing T with L in the subscripts of Eqs 17–20. To avoid confusion, we denote the fraction of ligand in the 1:1 state (corresponding to F_21_) as F_L,21_. Mathematically, the final fraction from the two-step process appears as

$$2T_{0}F_{T}\left(1-F_{T}\right)\left(1-F_{21}\right)\frac{2F_{L}\left(1-F_{L}\right)\left(1-F_{L,21}\right)}{2F_{L}\left(1-F_{L,21}\right)}=2T_{0}F_{T}\left(1-F_{T}\right)\left(1-F_{21}\right)\left(1-F_{L}\right),$$ (22)

where F_L_, the fraction of bivalents bound to target, excluding the 1:1 state, is given by an equation similar to Eq 21,

$$F_{L}=\frac{T_{0}}{L_{0}}\frac{R-F_{21}}{1-T_{0}F_{21}/L_{0}}.$$ (23)

Noting that T_0_<<L_0_ and that both R and F_21_ are less than or equal to 1, F_L_ is approximately 0. To find the fraction of targets with singly bound bivalent to singly bound target times the fraction which does not form the 1:1 state, multiply Eq 22 (in the limit of no 1:1 state possible) by (1-F_T,Dim_). The fraction F_T,Dim_ of singly bound target and bivalent which forms a 1:1 complex is further defined below. The limit of no 1:1 state is simply obtained by setting F_21_ to 0 in all the above equations. Setting the two parts equal gives

$$2T_{0}F_{T}\left(1-F_{T}\right)\left(1-F_{21}\right)=2T_{0}R\left(1-R\right)\left(1-F_{T,Dim}\right),$$ (24)

which, when solved for F_T_, yields

$$F_{T}=R\left(1-F_{T,Dim}\right).$$ (25)

Eq 25 intuitively makes sense, as the portion of the total bound sites which isn’t in the 1:1 state should be given by F_T_. Equating Eqs 25 and 21 and solving for F_21_ produces

$$F_{21}=\frac{RF_{T,Dim}}{1-R\left(1-F_{T,Dim}\right)}.$$ (26)

Only the determination of F_T,Dim_ remains. Most treatments assume that the tether is sufficiently long to permit simultaneous occupancy of both target sites by a single bivalent. We also consider cases where this assumption does not hold, since several experimental attempts, such as bivalent inhibitors of gyrase[30], have resulted in effectively monovalent inhibition profiles, either because the selected tether length did not permit bridging between the binding sites or other constraints prohibited a single bivalent from occupying both of the adjacent binding pockets. In these cases, the EC_50_ improves two-fold over the EC_50_ of the monomeric binder, single binding site case and closely parallels the bivalent binder, single binding site case and F_T,Dim_ = 0.

**Fig 2:**
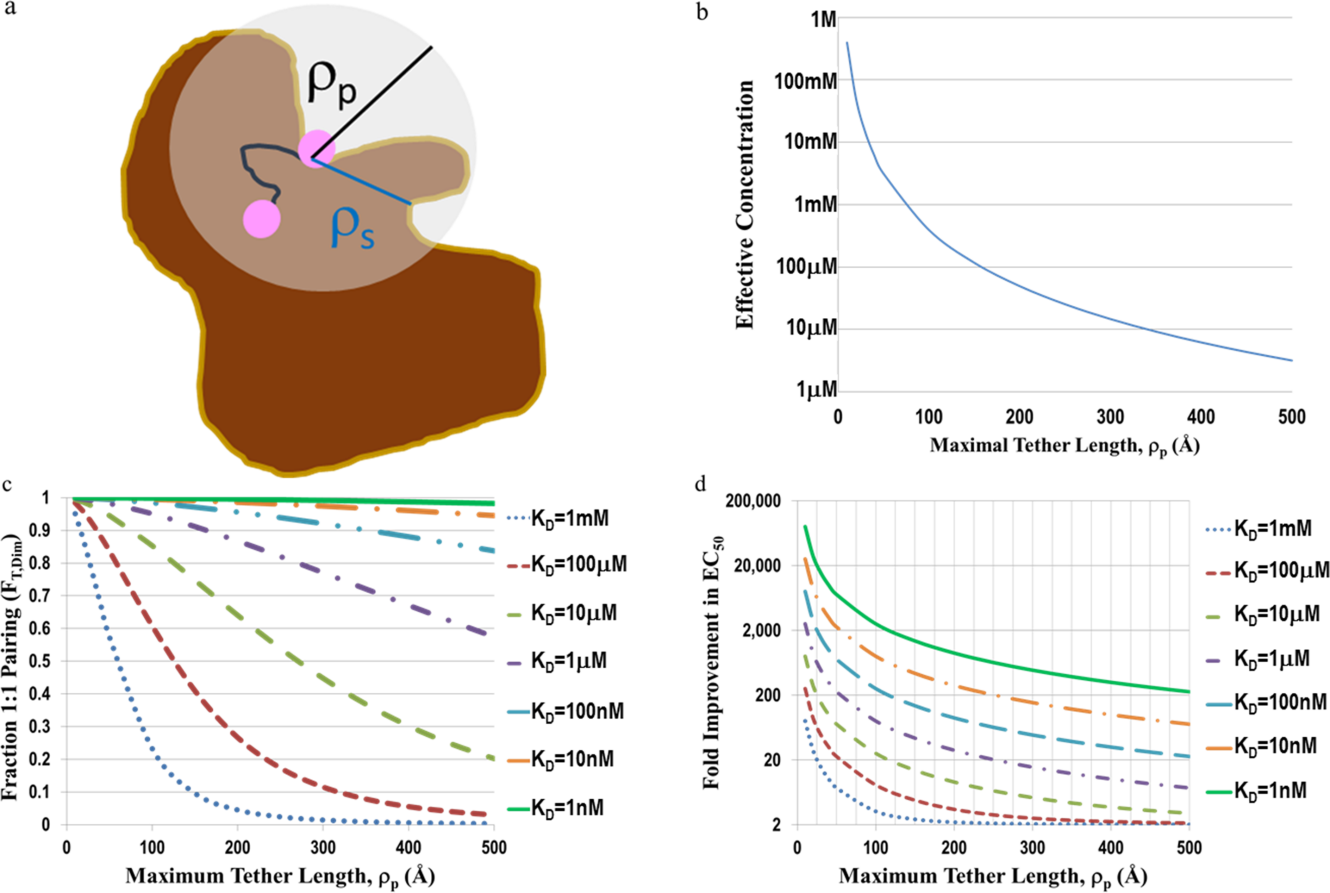
Significant increases in potency are possible for irreversibly connected monomers over a wide range of tether lengths. a) Model of the concentrating effect afforded by a tether. The target (brown) has two binding sites with a separation distance of *ρ*_*s*_ (blue line). One of the dimer’s ligands (pink) binds one target site while the other ligand is free in space. The fully extended dimer has a separation length *ρ*_*p*_ (black) between ligands. The sphere which encloses the space available to be sampled by the free ligand is grey. b) Semi-log plot of the effective concentration as a function of tether length from Eq 30 with *ρ*_*p*_ ≥ *ρ*_*s*_ c) Fraction of total target molecules which have both sites occupied by a single bivalent ligand (F_T,Dim_) as a function of tether length and dissociation constant for the monovalent ligand (K_D_) from Eq 28. d) Semi-log plot of fold improvement in the predicted EC_50_ for the bivalent ligand, two site target model relative to the predicted EC_50_ from the monovalent, single site target model as a function of tether length and K_D_.

If the connector is long enough, the first ligand will bind to one binding site of the empty target and permit the second ligand to access the second binding site. The protein, connector, and the first bound ligand are all assumed not to interfere with the ability of the second ligand to find the second binding site. Hence the second ligand can be treated as sampling a sphere whose radius is the maximum separation distance *ρ*_*p*_ between the ligands as determined by fully extending the tether (Fig 2A). This fully extended distance can be estimated by modeling software and is usually given in angstroms. The effective molar concentration L_0,eff_ of this ligand in this sphere is given by

$$L_{0,eff}=396.4/\rho_{p}^{3},$$ (27)

where *ρ*_*p*_ is in units of angstroms.

Assuming the macroscopic target concentration is more dilute than the microscopic ligand concentration, then, the effective concentration of sites within the same sphere is effectively just the one unoccupied binding site. Hence T_0,eff_ is also given by the right hand side of Eq 27. In this microcosm, the monomeric ligand, single binding site system is recapitulated, but with the effective starting concentrations as in Eq 27. Eq 4 therefore holds, with F_T_ in this case being the fraction of time the second ligand occupies the second binding site, which equals F_T,Dim_. Solving for F_T_ in Eq 4 yields

$$F_{T,Dim}=1-K_{D}/2L_{0,eff}\left[{\left(1+4L_{0,eff}/K_{D}\right)}^{1/2}-1\right].$$ (28)

Eq 28 confirms that as L_0,eff_ goes to infinity, F_T,Dim_ goes to 1; as L_0,eff_ goes to 0, F_T,Dim_ also goes to 0. The distance at which the effective ligand concentration equals that of the bulk is found by setting L_0,eff_ to L_0_ in Eq 27 and solving for *ρ*_*p*_,

$$\rho_{p}=7.346/L_{0}^{1/3},$$ (29)

suggesting that, for most micromolar affinity or better ligands, an enhancement in second ligand concentration is achievable for any tether length <100Å, subject to the assumptions of this model. The effective concentration at which the populations of 1:1 and 1:2 species are equal (F_T,Dim_ = ½) is given by L_0,eff_ = 2K_D_. To summarize, F_T,Dim_ is given by Eq 28 with

$$L_{0,eff}=\left\{ \begin{array}{l} 0,\rho_{p}<\rho_{s}\\ \frac{396.4}{\rho_{p}^{3}},otherwise \end{array}\right.,$$ (30)

where *ρ*_*s*_ is the distance between sites. Hence, all variables are now functions of the experimentally determinable R and of F_T,Dim_, which is a function of maximal spanning tether length, a constant for a given bivalent.

Regardless of what equilibrium condition for a sequential addition is considered, K_D_ is given by

$$K_{D}=2\frac{1-F_{T}}{F_{T}}\left(L_{0}-T_{0}R\right).$$ (31)

Using Eq 25, T_0_<<L_0_, and R = ½, Eq 31 is well approximated by

$$K_{D}=2\frac{1+F_{T,Dim}}{1-F_{T,Dim}}L_{0}.$$ (32)

Comparison of the EC_50_s from the monomeric ligand, single site model (Eq 7) and for this model (Eq 32) produces

$$EC_{50,bb}\approx EC_{50,mm}\left(1-F_{T,Dim}\right)/\left[2\left(1+F_{T,Dim}\right)\right].$$ (33)

Fig 2 shows a plot of the relationship between tether length and effective ligand concentration (Fig 2*B*), fraction in 1:1 complex (Fig 2*C*), or the effective fold decrease in EC_50_ relative to the monomeric ligand, single site model’s predicted EC_50_ (Fig 2*D*). These plots are only applicable when the maximal tether length exceeds the distance between the two sites.

Two immediate conclusions emerge. First, at short tether lengths, very large improvements in EC_50_ over the monovalent ligands are possible, provided the connectors can span the two binding sites. Second, with higher ligand affinity, the multiplier increases for a given tether length. This makes sense as intuition suggests binding to the two sites might scale as K_D_^2^ and hence the multiplier should scale as K_D_. Yet inspection of Fig 2*C* at R_p_ = 125Å suggests that two orders of magnitude change in K_D_ is necessary to effect a one order magnitude change in the multiplier. In order to understand this phenomenon, consider the scenario when L_0,eff_>>K_D_. Then Eq 33 becomes approximately

$$EC_{50,bb}\approx EC_{50,mm}{\left(K_{D}/L_{0,eff}\right)}^{1/2}/4,$$ (34)

thus explaining the dependence of the multiplier on the root of K_D_.

We also evaluated the scenario which allows the dissociation constant K_2_ for the equilibrium between the 1:1 state and the completely unbound ligand and target to scale as K_D_^2^ in this model. Solving for K_2_ yields

$$K_{2}=K_{D}\frac{1-F_{T,Dim}^{2}}{4F_{T,Dim}},$$ (35)

which implies that for K_2_ to scale as K_D_^2^,

$$K_{D}∼\frac{1-F_{T,Dim}^{2}}{4F_{T,Dim}}.$$ (36)

Since F_T,Dim_ is nearly 1, set F_T,Dim_ = 1–2 Δ, with Δ<<1, and use an equality in Eq 36 to obtain the scaling condition

$$K_{D}\approx \Delta ,$$ (37)

which satisfies the constraints on Δ even for millimolar affinity ligands. But from Eq 28,

$$K_{D}/2L_{0,eff}\left[{\left(1+4L_{0,eff}/K_{D}\right)}^{1/2}-1\right]=2\Delta \approx 2K_{D}.$$ (38)

Solving for L_0,eff_ gives

$$L_{0,eff}\approx \frac{1}{4K_{D}},$$ (39)

which implies (from Eq 27) that *ρ*_*p*_ ≈1.2Å if K_D_ = 10^-3^M, and even shorter if K_D_ is smaller. One may interpret this result to say that only a single bond between the ligands permits the intuitive scaling as the square of the individual ligands, with entropy costs diluting effective potency. Although it has an alluring qualitative appeal, this interpretation is dangerous because several of the key assumptions, including lack of influence of one ligand’s binding to the binding of the other and full sampling of a sphere of such short radius, are almost certainly violated in practice. Nevertheless, this result implies K_2_ should never be expected to scale as K_D_^2^ experimentally unless other interactions contribute. To get the general scaling of K_2_ with respect to K_D_ for tethers with lengths between 5 and 100Å, we substituted Eq 35 with the definition of F_T,Dim_ (Eq 28) and took the limit where L_0,eff_>>K_D_ to find

$$K_{2}\approx {\left[K_{D}^{3}/4L_{0,eff}\right]}^{1/2}.$$ (40)

Hence, K_2_ scales as the 3/2 power of K_D_ and not the second power in this limit.

Fortunately, studies on synthetic bivalent inhibitors of carbonic anhydrase[17] have generated clean data expected to fall under this model. In this experimental system, the tethers do not affect the affinity of the ligands. The monovalent ligand “Compound 6” had an observed ΔG of -9.6 kcal/mol. Based on the scaling arguments above and the affinity of compound 6, the affinity of the corresponding bivalent should be around -14.4 kcal/mol, which is, in fact, close to the observed affinity of -15.7 kcal/mol. Further, dependence on chain length was weak—a rough doubling of the fully extended connector length led to a 1–1.5 kcal/mol loss in affinity, qualitatively in line with the current model. Finally, the model predicts a 450 to 1600 fold improvement in potency, once again in qualitative agreement with the observed 400 to 5300 fold improvement. These experiments show a dip in potency at shorter tether lengths, posited in the paper to arise from particular interactions of the tether with the protein. Based on the model described here, deviations from ideality are most likely due to loss of favorable protein contacts at shorter tether lengths or, less likely, due to gain of favorable protein contacts at particular tether lengths. Since the protein does not remain rigid but flexes, it may break additional contacts when constrained by short tethers, influencing the overall affinity.

Similar data was generated in studies of the activity of cGMP homodimers linked with polyethylene glycol (PEG) [31]. When tested against rat olfactory cyclic-nucleotide-gated (CNG) channels, a 5000Da PEG linker attached to a single cGMP increased activity by two fold over cGMP itself (1.4 vs. 3.1 μM). Making a bivalent with too short a linker [282PEG-(cGMP)_2_] only further improved the activity by a factor of two (0.87 μM), in line with this model. 2000Da of PEG linker gave the maximal increase in activity (~200-fold), well below the current model’s prediction (845-fold). Based on the data, the model predicts this maximum to occur around 2200Da of PEG. Though showing the right qualitative trend, the reported decrease in activity for linkers going from 3400 to 20,000Da of PEG (~21-fold loss) varied from that predicted from the model (~360-fold loss), suggesting the possibility that 1) more potent inhibitors may exist with linkers between 2000 and 3400Da and 2) the PEG linker serves to positively increase binding affinity. When the homodimers were tested against rod photoreceptor CNG channels, their affinity is predicted to be no better than ~600 nM, since cGMP affinity is only 72 μM. This prediction is in line with the observed ~450 nM for a 1200Da linker and ~550 nM for a 2000Da linker. The increase in linker from 2000Da to 3400Da results in an observed affinity of ~5μM, consistent with the predicted lowered affinity of ~2.7 μM.

The model can also be used to provide upper-bound limits on expected activity in a cellular context. For example, the (6+2) homodimers of JQ-1 showed BRD4 inhibition as low as 220 pM in cellular assays, as compared to ~70 nM activity for JQ-1 in the same cellular assays[12]. Based on the model, a 70 nM inhibition should lead to no more than about 20 pM inhibition from the dimers. Hence the model implies that either the optimal linker distance was not found or the dimer struggles to penetrate the cell membrane relative to JQ-1. In another example, homodimers of a modified oxytocin (dOTK_2_-C8) activated the oxytocin receptor dimers at 0.8 pM concentrations in cellular assays, as compared to 4 nM concentrations for the monomer, dOTK [32]. Based on the model, 4 nM monomer activity translates into ~0.25 pM activity for the best dimers, in line with their observations.

Similarly, Andersen et al. [33] looked the affinity of homodimers of either serotonin (5HT) or dopamine (DA) towards either serotonin (SERT) or dopamine (DAT) transporters. Making this study more interesting, they employed either alkyl or PEG linkers which had differential effects on the potencies. PEG linkers hardly affected 5HT activity against either transporter, whereas it improved DA activity towards SERT but damaged activity against DAT. Nevertheless, all the dimers with the greatest affinities for a given transporter and ligand came close to the theoretical “best attainable” values from this model, except for the DA dimer against SERT. In this case, the activity observed was ~10-fold less than the best predicted, suggesting the linker could not provide the full enhancement given when only one DA was present. The alkyl linkers, when attached to either ligand, produced large affinity boosts, which then failed to translate into extremely more potent dimer affinities when the second ligand was attached. This result likely indicates membrane incorporation by the linker which facilitates binding to the transporters. Indeed, the best affinities observed came much closer to those predicted by the model using the affinities of the ligands alone. These examples warn against blind application of this model without consideration of the experimental conditions and how the linkers may behave under those conditions.

### Dual binding sites, reversibly linked monomers/dimers

**Fig 3:**
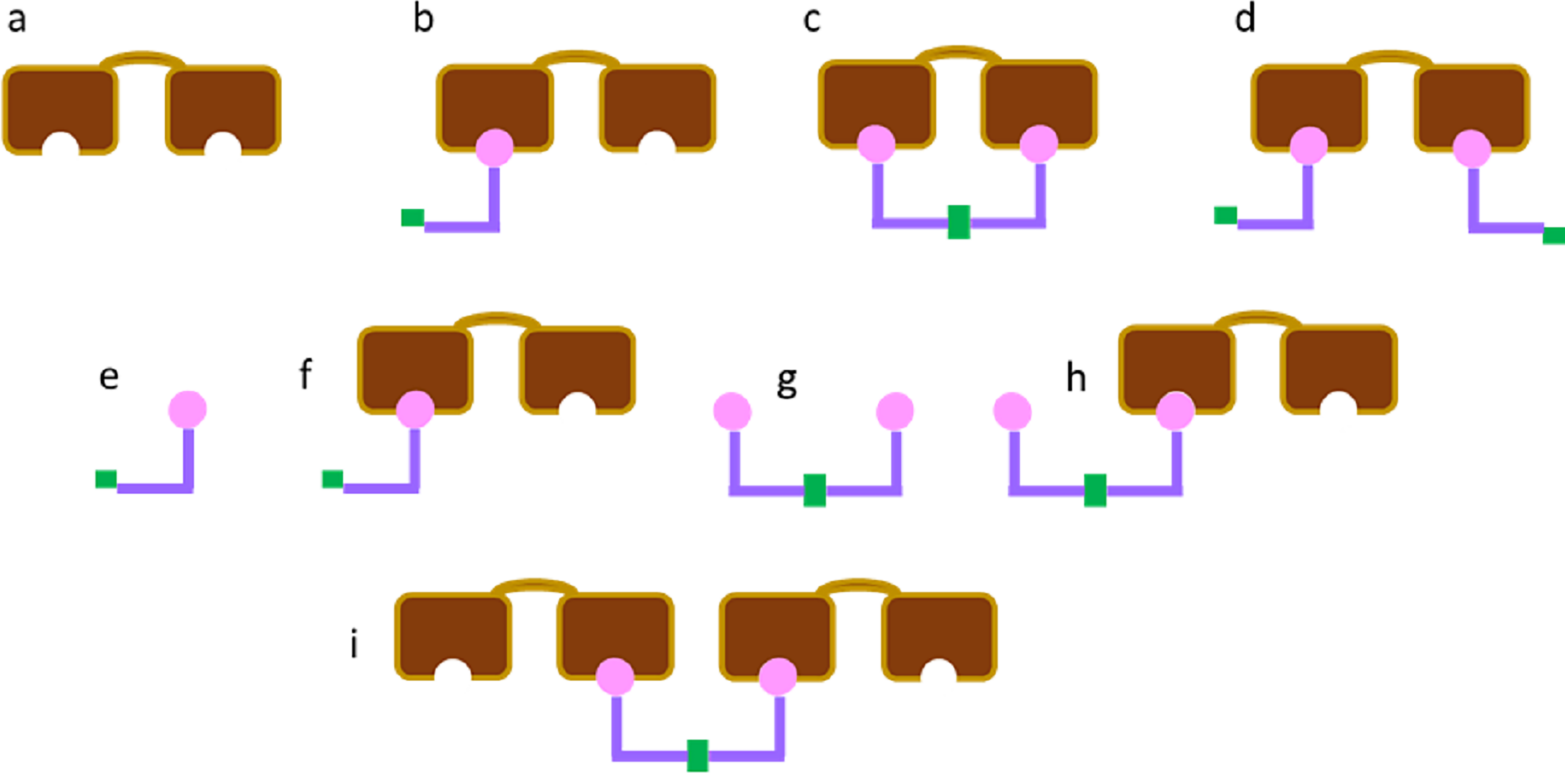
Schematic representation of the possible interactions between two site targets and dimerizing monomers. The green bar represents the linker (i.e., the dimerization moiety) attached to a ligand (pink). For calculating target occupancy probabilities, a single target (brown) may have no monomer bound (a), only one monomer bound (b), or two monomers bound. For the latter scenario, the two monomers could dimerize (c) or lack any association with each other (d) on a single target. Whenever a ligand is depicted as undimerized in (b)-(d), it is done so as a convenience, since its dimerization state is irrelevant to determining the probability of site occupancy on a single target molecule. For calculating ligand occupancy probabilities, occupancies for both the monomeric and dimeric states must be included. A single monomer may have its ligand bound (f) or not (e). A single dimer can have no ligands bound (g), one ligand bound (h), or two ligands bound. When two ligands are bound, they could share the same target (c) or be bound to separate target molecules (i). Whenever a target is depicted as having an unbound site in (f)-(i), it is done so as a convenience, since the occupancy of its other site is irrelevant to determining the probability of site occupancy on a single ligand.

One potential benefit of dimerizing monomers over pre-assembled irreversibly linked analogs is that they can act as monomers with respect to cell penetration and as dimers with respect to target affinity. In this model, the dimerizing moieties (or linkers) responsible for reversible tethering are treated as identical single points on the distal end of a completely flexible connector extending from the ligand. A significant number of species have to be considered in this case, each of which are illustrated in Fig 3. More compact mathematical descriptions of the various species may be required for systems of any greater complexity [34, 35].

Let F_u_ be the fraction of completely unbound target (Fig 3*A*), F_1_ the fraction of singly bound targets (Fig 3*B*), F_21_ the fraction of 1:1 state (Fig 3*C*), and F_22_ be the fraction of doubly bound target not in the 1:1 state (Fig 3*D*). As in the case of irreversibly linked ligands, the species depicted in Fig 3B and 3D, feature contributions from both monomeric and dimeric ligand bound. Further, let L_u_ be the fraction of unbound monomer (Fig 3*E*), L_1_ the fraction of bound monomer (Fig 3*F*), D_u_ the fraction of unbound dimer (Fig 3*G*), D_1_ the fraction of singly bound dimer (Fig 3*H*), D_21_ the dimer fraction in the 1:1 state (Fig 3*C*), and D_22_ the fraction of doubly bound dimer not in the 1:1 state (Fig 3*I*). Intuitively, we can group L_u_ and L_1_ into the fraction of monomers, F_ML_. Although a similar grouping is possible for dimer, we wish to exclude the 1:1 state, as in the dual binding site, bivalent ligand model. Hence, we group only D_u_, D_1_, and D_22_ into the dimer fraction F_DL_.

The total number of sites must be conserved both for the targets,

$$1=F_{u}+F_{1}+F_{22}+F_{21},$$ (41)

and for the ligands,

$$1=F_{ML}+F_{DL}+D_{21}.$$ (42)

Much as in Eqs 17–19, of the fraction which is not bound in the 1:1 state, the distribution must behave as in the monovalent binder, dual binding site model, namely

$$F_{u}={\left(1-F_{T}\right)}^{2}\left(1-F_{21}\right),$$ (43)

$$F_{1}=2F_{T}\left(1-F_{T}\right)\left(1-F_{21}\right),$$ (44)

$$F_{22}=F_{T}^{2}\left(1-F_{21}\right),$$ (45)

where F_T_ is the fraction of targets sites not occupied by the 1:1 state but otherwise occupied by ligand. F_T_ is related to F_21_ through a relation identical to Eq 21. Similarly, excluding the 1:1 state, the monomeric fractions and dimeric fractions all behave as in the monovalent binder, single binding site and bivalent binder, single binding site scenarios, respectively, implying

$$L_{u}=\left(1-F_{L,M}\right)F_{ML},$$ (46)

$$L_{1}=F_{L,M}F_{ML},$$ (47)

$$D_{u}={\left(1-F_{L,D}\right)}^{2}F_{DL},$$ (48)

$$D_{1}=2F_{L,D}\left(1-F_{L,D}\right)F_{DL},$$ (49)

$$D_{22}=F_{L,D}^{2}F_{DL},$$ (50)

where F_L,M_ and F_L,D_ are the fractions of the monomer and dimer populations, respectively that occupy binding sites on the target. F_L,M_ and F_L,D_ can be related by comparing the dissociation constants for completely unbound monomer or dimer binding to completely unoccupied target,

$$\frac{T_{0}F_{u}L_{0}L_{u}}{\left(1/2\right)T_{0}F_{1}L_{1}/\left(F_{L,M}F_{ML}+F_{L,D}F_{DL}\right)}=K_{D}=\frac{T_{0}F_{u}L_{0}D_{u}}{\left(1/2\right)T_{0}F_{1}\left(D_{1}/2\right)/\left(F_{L,M}F_{ML}+F_{L,D}F_{DL}\right)}.$$ (51)

Simplifying the right and left hand sides of Eq 51 and substituting in Eqs 46 to 50 permits reduction to

$$F_{L,M}=F_{L,D}\equiv F_{L}.$$ (52)

Eq 52 is unsurprising, given that the target cannot distinguish whether the binder is associated with a monomer or a dimer. F_L_ can be related to R’, the total fraction of monomers bound to target sites, which itself can be related to R,

$$R'=R\frac{2T_{0}}{L_{0}}=F_{L}\left(1-D_{21}\right)+D_{21}.$$ (53)

Since L_0_D_21_ = 2T_0_F_21_, Eq 53 may be solved for F_L_ to yield

$$F_{L}=\frac{2T_{0}\left(R-F_{21}\right)}{L_{0}-2T_{0}F_{21}},$$ (54)

suggesting that, once again, F_L_ is well approximated by 0 whenever L_0_>>T_0_. F_DL_ may be rendered a dependent variable by recognizing that

$$K_{Dim}=2L_{0}\frac{F_{ML}^{2}}{F_{DL}},$$ (55)

where K_Dim_ is the dimer dissociation constant for the ligand and the factor of 2 comes from the fact that the dimer concentration is half that of the monomers which comprise them. Substitution of Eq 42 into Eq 55 and solving for F_DL_ yields

$$F_{DL}=1-D_{21}-\frac{K_{Dim}}{4L_{0}}\left\{{\left[1+\frac{8L_{0}}{K_{Dim}}\left(1-D_{21}\right)\right]}^{1/2}-1\right\}.$$ (56)

Much as in the bivalent ligand model, the remaining independent variable, F_21_, can be resolved by using the two step thermodynamic process. Since the formation of 1:1 state can be achieved either by dimerization of bound monomeric ligands or by binding of the second ligand of a preformed dimer which is already bound to one site, one can solve for F_21_ in the limit of either extreme and then, for the general case, use a fractional contribution of each to achieve the desired final expression for F_21_. The fractions are derived from the portions of monomeric ligand bound to two binding sites on a single target (the monomeric limit) and of a dimer bound to one binding site of the target, with the other binding site open and the other monomer unbound (the dimeric limit). Clearly this solution has the appealing benefits of being nearly exact when the reversibly linked monomers are either largely monomeric or largely dimeric in solution and provides an intuitive feel for behavior when the starting concentration is within an order of magnitude or so of the dimerization constant.

For the dimeric limit, it is possible to show that all the results from the previous section apply here, with the understanding that the maximum tether length in the previous section is twice the length of the monomer connector length and the concentration in the previous section is half that of the starting monomeric concentration in this section. In the monomeric ligand limit, the two step thermodynamic equivalence is

T0F22(L1FL(1−D21))2=T0R2(L1'FL)2(1−FL,Dim), (57)

where F_L,Dim_ is the fraction of monomers which dimerize on the target, an expression which will be derived in more detail below, and L_1_’ is L_1_ evaluated in the limit of D_21_ = 0. Solving for F_21_ in Eq 57 produces

$$F_{21}=R\left\{1-R\frac{1-F_{L,Dim}}{2}-{\left[R^{2}{\left(\frac{1-F_{L,Dim}}{2}\right)}^{2}+\left(1-R\right)\left(1-F_{L,Dim}\right)\right]}^{1/2}\right\}.$$ (58)

The derivation of F_L,Dim_ follows closely from the derivation used for the bivalent ligand scenario described earlier. If the linkers between monomers are too short to permit dimerization, F_L,Dim_ = 0. If the linkers are long enough, we considered first the situation in which both monomers have bound to a single target. The protein, connectors, and bound monomers are all assumed not to alter the ability to dimerize. Hence the moieties responsible for dimerizing can be treated as each sampling a sphere whose radius is half the maximum separation distance *ρ*_*p*_ between the ligands. When the separation between ligands is exactly the distance between target sites, the effective concentration L_0,eff_ of each dimerization moiety is given by

$$L_{0,eff}=\frac{396.4}{{\left(\rho_{p}/2\right)}^{3}}=3171.2/\rho_{p}^{3},$$ (59)

where *ρ*_*p*_ is in units of angstroms. The effective concentration when the maximum separation is much larger than the separation between target sites is essentially twice that of the right hand side of Eq 59 since the two spheres nearly entirely overlap. Clearly, between those two limits features a factor between 1 and 2 to multiply the right hand side of Eq 59. For the current analysis, Eq 59 will be used regardless of the relative magnitudes between maximum separation and distance between target sites, provided the maximum separation can at least span the two sites, in order to focus on the qualitative trends. Using Eq 55 and defining the effective ligand concentration as in Eq 59 produces the desired expression for F_L,Dim_,

$$F_{L,Dim}=1-\frac{K_{Dim}}{4L_{0,eff}}\left[{\left(1+\frac{8L_{0,eff}}{K_{Dim}}\right)}^{1/2}-1\right].$$ (60)

The ratio of the fractions of targets with 2 monomers bound to those having one otherwise unbound dimer should, in the limit of F_21_ = 0, provide a reasonable estimate of the proportionate contributions from the two extremes to F_21_ values given by Eqs 26 and 58. Put another way,

$$\begin{array}{l} F_{21}\approx \frac{RF_{ML}^{2}}{RF_{ML}^{2}+2\left(1-R\right)F_{DL}}R\left\{1-R\frac{1-F_{L,Dim}}{2}-{\left[R^{2}{\left(\frac{1-F_{L,Dim}}{2}\right)}^{2}+\left(1-R\right)\left(1-F_{L,Dim}\right)\right]}^{1/2}\right\}+\\ \frac{2\left(1-R\right)F_{DL}}{RF_{ML}^{2}+2\left(1-R\right)F_{DL}}\frac{RF_{T,Dim}}{1-R\left(1-F_{T,Dim}\right)}. \end{array}$$ (61)

The relevant expression for K_D_ at R = ½ (see equation S48 in the supplemental material) is

$$\frac{L_{0}}{1-2F_{21}}=K_{D}.$$ (62)

with the denominator of Eq 62 being

$$1-2F_{21}=\frac{{\left(1-F_{DL}\right)}^{2}}{F_{DL}^{2}+1}\left\{\frac{1-F_{L,Dim}}{4}+{\left[{\left(\frac{1-F_{L,Dim}}{4}\right)}^{2}+\frac{1-F_{L,Dim}}{2}\right]}^{1/2}\right\}+\frac{2F_{DL}}{F_{DL}^{2}+1}\left(\frac{1-F_{T,Dim}}{1+F_{T,Dim}}\right).$$ (63)

**Fig 4:**
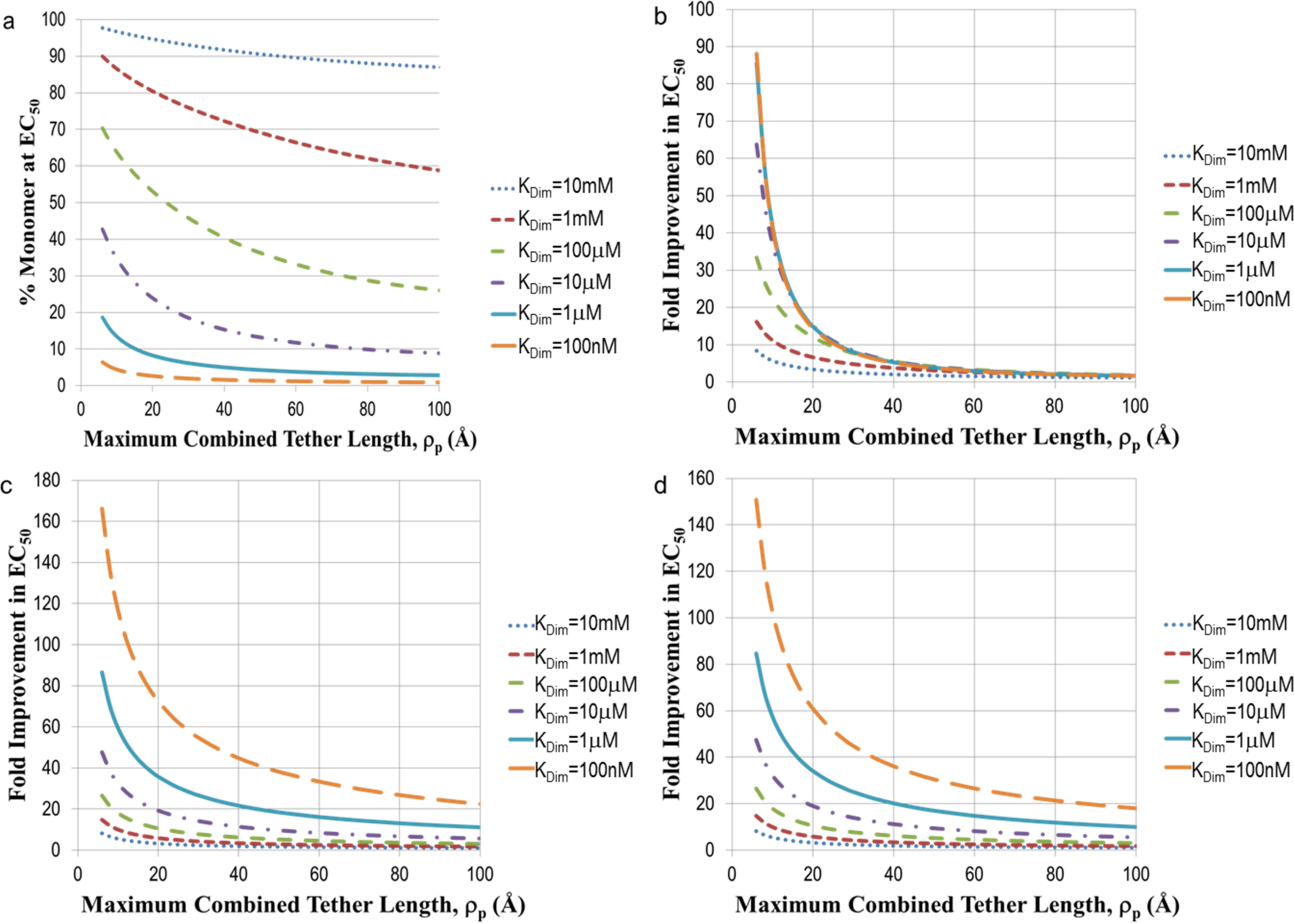
Significant increases in potency are possible for reversibly connected monomers over a wide range of tether lengths and monomer affinities. A) Fraction of monomer in solution at a starting concentration equal to the EC_50_, as a function of dimerization constant K_Dim_ and tether length *ρ*_*p*_, when the K_D_ of the monomer is 1 mM. In the remaining insets, fold improvement in the EC_50_ relative to that for a single site target, single ligand system is plotted as a function of the dimerization constant and tether length with K_D_ = 1 mM B), K_D_ = 1 μM C), or K_D_ = 1 nM D).

Substituting Eq 63 into Eq 62 permits evaluation of the fold improvement of the EC_50_ over that for the monomeric non-dimerizing ligand, single binding site scenario. Since F_DL_ is a function of L_0_, the solutions are too complex for a closed form expression and the data are generated from solving Eq 62 numerically for L_0_, given a particular value of K_D_, K_Dim_, and *ρ*_*p*_. Fig 4 illustrates results for a few different scenarios. When the ligands themselves are extremely weak, the fraction of monomer and dimer is a sensitive function of the dimerization constant. With very weak dimerization constants (i.e., K_Dim_>K_D_), the EC_50_ is influenced primarily by the presence of monomer, while more potent dimerization constants (i.e., K_Dim_ <K_D_) feature EC_50_’s driven primarily by dimer (Fig 4*A*). Hence the calculation of the fold-improvement (Fig 4*B*) produces largely overlapping curves when the dimerization constants are much smaller than those for the dissociation constant of the ligand-target complex. When the dimerization and affinity constants are nearly equal, the dependence is a sensitive function of the tether length. Note that the magnitude of the fold-improvement is almost exactly half that of the dimer case when the dimer dominates the contribution, since the concentration of dimer formed from monomer is half the starting concentration of monomer. An experimentally meaningful difference of 4-fold is detectable even when the dimerization constants are very weak, namely for *ρ*_*p*_ <16Å when K_Dim_ = 10^-2^M and for *ρ*_*p*_ <~40Å when K_Dim_ = 10^-3^M. These results are encouraging since detection of weak ligands is challenging. Adding reversible linkers allows rapid exploration of appropriate linker lengths in small synthetic libraries, while increasing the ability to detect target modulation through improved potencies. Further, dimerizing monomers with relatively short linkers and good dimerization constants permits the transformation of fragment-based screening hits (with K_D_≥~10^-4^M) into potential tools for cell based assays with no further optimization. This aspect was well demonstrated using Myc inhibitors, in which the ligands had weak activity in cells, even in combination, but their reversibly linked analogs had low micromolar activities[27].

By the time the ligand affinity reaches that of typical screening hits (K_D_~10^-6^M), the majority of the fold improvement is driven by target-associated dimerization of monomers and not by binding of dimer preformed in solution. Even at K_Dim_ = 100nM, *ρ*_*p*_ would have to extend out to almost 250Å before finding equal contributions from monomer and dimer in solution. Effectively, for almost any dimerization constant and for all *ρ*_*p*_ ≤100Å, an EC_50_ ≤~25μM is predicted to have at least 50% contribution from target-driven dimerization of monomers. Under these conditions, dimerization of micromolar binders allows affinities to be driven into the low to mid nanomolar range (Fig 4*C*). In the final scenario of very potent ligands, the fold improvements are still similar to those observed for weaker ligands (Fig 4*D*), since monomer present in solution entirely drives the observed effects.

## Conclusion

We have developed computational models to describe the thermodynamics of reversibly and irreversibly tethered homodimeric molecules binding to targets with dual binding sites. In these models, we have considered the simplest systems of dimers, connected with completely flexible tethers that interact with rigid targets containing two identical binding sites. In the simpler scenario, the tether is irreversibly connected. These irreversibly linked compounds have been investigated in recent years [15–22]; however, the length dependence of the tether on compound affinity has not been well treated theoretically. We developed a model to treat the length dependence and found that short tethers, which enable bivalents to simultaneously occupy both sites, can induce very significant fold improvements (>10,000x) over their monomeric counterparts. Avidity primarily drives these large improvements in effective binding, with extremely high (molar) effective concentrations of monomers generated for a second site when a first monomer is bound to a target. The predicted effective concentration follows an inverse cubic dependence on tether length, implying that roughly an order of magnitude in effective concentration is lost with each doubling in maximal spanning distance between ligands. Nevertheless, at a separation of 10Å, the predicted effective concentration is around 0.5M. Hence typical monovalent ligand concentrations (~1 μM) are not seen until >300Å maximal separation between the ligands of the bivalent. This relatively rapid flattening of the effective concentration curve suggests that, for all but the weakest monovalent ligands, the fold improvement in binding of the bivalent to the target only weakly depends on tether length for most contemplated compounds in pharmaceutical research [17], with the most variation in fold improvement seen among tethers of shortest length.

The predicted effective scaling of the dissociation constant for bivalent compounds binding to targets with two binding sites is perhaps somewhat unexpected. Intuition might suggest that the easiest estimate of the affinity gain should be given by a doubling of the free energy of binding since each ligand binds independently of the other to each site, thus each contributing the same free energy of binding in a single molecule. Jenks and others [36, 37] anticipated that this should never be the case, yet many persist in using this doubling as a “zeroth order” approximation. Converting free energies into dissociation constants, the predicted effective dissociation constant should therefore go as K_D_^2^, rather than as the dissociation constant K_D_ for the monomeric ligand. The same reasoning should apply to the binding of such molecules even when the tether between them becomes infinitely long. Yet, in this limit, the bivalents should behave essentially as monomeric ligands, for which the effective dissociation constant is the actual dissociation constant for the monomer, independent of how many sites are on the target. Since the concentration of bivalent is half that of the monomer to yield the same EC_50_, a mere two fold improvement in effective K_D_ is predicted for bivalents in this limit of infinite tether length. Our model corrects for this straight additivity problem by explicitly treating the entropic and energetic changes imposed on the binding of the second monomer by the binding of the first monomer. Our model suggests the effective dissociation constant should scale as K_D_^3/2^, whenever tether lengths are sufficiently long (>5Å) but not overly so (typically <100Å, above which the effective ligand concentration becomes similar to the monomer dissociation constant).

This prediction of the effective dissociation constant going as the 3/2 power of the ligand dissociation constant matches the experimental data with bivalent carbonic anhydrase inhibitors [17]. The experimental data in that report imply that the tethers do not influence monomer affinity, making their studies compatible with this analysis. Those bivalents generally grew weaker with increasing tether length, but only modestly so, and their free energies were roughly 1.5 times as large as those for the corresponding ligands. Other near ideal experiments also support this scaling behavior, as illustrated in the cases of cGMP homodimers binding to various dimeric receptors, homodimers of JQ-1 binding to BRD4, homodimers of a modified oxytocin binding to the oxytocin receptor, and homodimers of either 5HT or DA binding either SERT or DAT transporters [12, 31–33]. The 3/2 power scaling of the monomer’s dissociation constant should be used as the “zeroth order” approximation of the dissociation constant for most irreversibly linked dimers.

This halving of the free energy contribution from the second ligand may be understood as the entropic cost of effectively not allowing the second ligand to sample any potential target sites except for the other binding site on the same target molecule. Once the tethers are sufficiently long, the second ligand can sample more potential target sites but the fractional contribution from the second ligand *decreases* (down to nearly zero at infinite connector length) due to the increasingly weak contribution of a single ligand in a large volume of dilute targets. Although the model also predicts a greater fraction of contribution from the second ligand at shorter distances (i.e., at maximum tether lengths shorter than 5-10Å), it is likely that the rotational energy constraints on the tether reduce the second ligand’s ability to sample the entire sphere, resulting in a greater chance of overestimating fold improvement at these shorter distances.

While this model is simple, it is expected to describe corresponding experimental systems fairly well. For example, we predict the assumption of rigid target site separation is a weak constraint. Most protein active sites move but do not undergo large conformational rearrangements upon ligand binding. Based on this fact, the separation can be recast as the separation between sites averaged over all low energy protein conformations. If structure is being used to select tether lengths, a variety of tether lengths around the modeled optimum should be considered to allow for the protein movement.

We are working on extending this model to include allosteric effects or other asymmetric affinities for the two sites, especially since the second site can provide a mechanism for selectivity. Further, this extension of the model is critical as most dual site binding molecules have significantly different compositions, as well as affinities, and may be modeled poorly using the current model. Nevertheless, the current model can be used to provide a guide for expected changes in effective dissociation constant in most cases where the affinity for the two sites is similar, regardless of chemical differences. Large deviations from values predicted by the model suggest significant microscopic interactions, such as sterics or tether cooperativity, are at play. Taking the ratio between experimental and theoretical fold changes can serve as an easy guide for understanding microscopic contributions to the experimental data. Alternately, one may use more complex models[38, 39]. In general, to determine the fold improvements from this model, one needs the experimentally determined dissociation constant for the monovalent ligand without any connector as well as a theoretical calculation of the maximal separation between ligands in a given bivalent. To increase confidence that the model will predict bivalent behavior, a single ligand with a tether attached can be tested experimentally. Any changes in effective dissociation constant from tether addition should be included in the model’s predictions through treating the ligand and tether as a new ligand entity.

Given the very significant gains in effective target potency (and potentially selectivity) that bivalents offer, can bivalents represent the future of drug discovery? The current work explores the interaction of a bivalent molecule with a two-site target. While this is a fairly clean representation of what happens in some biophysical or biochemical assays, describing a bivalent’s ability to interact with cells or living organisms is decidedly more complex. In fact, the Rule of 5 suggests large molecular weight molecules are unlikely to become drugs. Many compounds which violate the molecular weight rule have difficulty permeating membranes. Those which do get into cells are typically macrocyclic. Even from those, many have difficulty being absorbed orally. All these aspects can be overcome, albeit with some effort, as the clinical agents for BCL-XL attest to [40].

In order to address some of these challenging aspects of irreversibly linked bivalents, monomers with reversible linker moieties may be employed. This work describes the interactions of such monomers with targets which contain two binding sites. Determination of the key values for deployment in the model is the same as those for the irreversibly linked bivalents, except that a dimerization constant for the monomers must be found as well. This constant can be found from model systems or from the dimerizing pair itself using physical methods such as NMR, LC/MS, or SPR. The model predicts that these monomers can result in significant improvements in effective dissociation constants over a broad range of connector lengths as well as dimerization and monomer-protein dissociation constants.

Although the literature features very little about small molecule monomers that can reversibly link to each other while binding identical target sites, nature frequently uses non-covalently interacting dimeric species to effect various regulatory functions. For example, various transcriptional factors can interact with DNA to increase transcriptional recruitment in certain regions. These interactions typically involve significant nuance based on, for example, post-translational modifications and small-molecule-protein interactions. While the current approach could, in principle, be applied to these interactions, it lacks the ability to describe the influence of these other factors which either significantly increase the number of interacting partners or involve inhibitors to dimer formation. Others [41, 42] have provided a detailed model for these cases.

Nevertheless, the approach to dynamically creating tethered small molecule monomers *in situ* takes advantage of the relatively fast on-rates of the monomers and of the relatively slow off-rates of bivalents. It allows for the intracellular generation of dimers, well suited for addressing “undruggable” biological targets, such as those with protein-protein interfaces, while capitalizing on the drug-like properties of the monomers. In solution, these monomers present as a rapidly interchanging mixture of monomers and dimers. Membrane permeation and oral absorption of monomers is generally more likely than their equivalent dimers and Le Chatlier’s principle suggests that, in the approach to equilibration across the membrane, more of the dimer will break up on the higher concentration side and reform on the lower concentration side, effectively driving dimer across membranes. Further, both the linkers and connectors can be tuned independently to achieve a desired pharmacokinetic profile. We speculate that full optimization is likely less critical, however, as the formation of high affinity dimers binding a specific target should improve the *in vivo* profile of both monomers when co-dosed, relative to their individual dosing profiles.

The approach is not altogether conceptual. Monomers based off well-established fragments and containing reversible linker moieties can serve as the basis of screening libraries, permitting rapid searches of chemical space for modestly potent and selective inhibitors. This enablement was well illustrated in the search for Myc-specific cell active inhibitors [27]. While the Myc monomers were distinct and targeted different sites, they were largely inactive in cell based assays. Reversibly linked versions of these monomers, on the other hand, had detectable activity correlated with Myc-specific activity. Hence an approach that incorporates reversible linkers has the potential to transform traditional drug discovery efforts, particularly against more challenging biological targets.

## Supporting information

S1 File This file contains derivations for some of the equations. (PDF)

## References

[1] Lipinski CA, Lombardo F, Dominy BW, Feeney PJ. Experimental and computational approaches to estimate solubility and permeability in drug discovery and development settings. Adv Drug Deliv Rev. 2001;46(1–3):3–26. 11259830 10.1016/s0169-409x(00)00129-0

[2] Kuntz ID, Chen K, Sharp KA, Kollman PA. The maximal affinity of ligands. Proc Natl Acad Sci U S A. 1999;96(18):9997–10002. 10468550 10.1073/pnas.96.18.9997 PMC17830

[3] Seco J, Luque FJ, Barril X. Binding site detection and druggability index from first principles. J Med Chem. 2009;52(8):2363–71. doi: 10.1021/jm801385d 19296650 10.1021/jm801385d

[4] Bakan A, Nevins N, Lakdawala AS, Bahar I. Druggability Assessment of Allosteric Proteins by Dynamics Simulations in the Presence of Probe Molecules. J Chem Theory Comput. 2012;8(7):2435–47. doi: 10.1021/ct300117j 22798729 10.1021/ct300117j PMC3392909

[5] Halgren TA. Identifying and characterizing binding sites and assessing druggability. J Chem Inf Model. 2009;49(2):377–89. doi: 10.1021/ci800324m 19434839 10.1021/ci800324m

[6] Nayal M, Honig B. On the nature of cavities on protein surfaces: application to the identification of drug-binding sites. Proteins. 2006;63(4):892–906. doi: 10.1002/prot.20897 16477622 10.1002/prot.20897

[7] Buchwald P. Small-molecule protein-protein interaction inhibitors: therapeutic potential in light of molecular size, chemical space, and ligand binding efficiency considerations. IUBMB Life. 2010;62(10):724–31. doi: 10.1002/iub.383 20979208 10.1002/iub.383

[8] Yu F, Peng Y, Wang Q, Shi Y, Si L, Wang H, et al Development of bivalent oleanane-type triterpenes as potent HCV entry inhibitors. Eur J Med Chem. 2014;77:258–68. doi: 10.1016/j.ejmech.2014.03.017 24650713 10.1016/j.ejmech.2014.03.017

[9] Haiying Sun LL, Lu Jianfeng, Bai Longchuan, Li Xiaoqin, Nikolovska-Coleska Zaneta, McEachern Donna, Yang Chao-Yie, Qiu Su, Yi Han, Sun Duxin, and Wang Shaomeng. Potent Bivalent Smac Mimetics: Effect of the Linker on Binding to Inhibitor of Apoptosis Proteins (IAPs) and Anticancer Activity. J Med Chem. 2011;54:3306–18. doi: 10.1021/jm101651b 21462933 10.1021/jm101651b PMC3108148

[10] Jahromi AH, Fu Y, Miller KA, Nguyen L, Luu LM, Baranger AM, et al Developing bivalent ligands to target CUG triplet repeats, the causative agent of myotonic dystrophy type 1. J Med Chem. 2013;56(23):9471–81. doi: 10.1021/jm400794z 24188018 10.1021/jm400794z PMC3925341

[11] Gower CM, Chang ME, Maly DJ. Bivalent inhibitors of protein kinases. Crit Rev Biochem Mol Biol. 2014;49(2):102–15. doi: 10.3109/10409238.2013.875513 24564382 10.3109/10409238.2013.875513 PMC4627631

[12] Tanaka M, Roberts JM, Seo HS, Souza A, Paulk J, Scott TG, et al Design and characterization of bivalent BET inhibitors. Nat Chem Biol. 2016;12(12):1089–96. doi: 10.1038/nchembio.2209 27775715 10.1038/nchembio.2209 PMC5117811

[13] Waring MJ, Chen H, Rabow AA, Walker G, Bobby R, Boiko S, et al Potent and selective bivalent inhibitors of BET bromodomains. Nat Chem Biol. 2016;12(12):1097–104. doi: 10.1038/nchembio.2210 27775716 10.1038/nchembio.2210

[14] Machida S, Kato N, Harada K, Ohkanda J. Bivalent inhibitors for disrupting protein surface-substrate interactions and for dual inhibition of protein prenyltransferases. J Am Chem Soc. 2011;133(4):958–63. doi: 10.1021/ja1086112 21158442 10.1021/ja1086112

[15] Monine MI, Posner RG, Savage PB, Faeder JR, Hlavacek WS. Modeling multivalent ligand-receptor interactions with steric constraints on configurations of cell-surface receptor aggregates. Biophys J. 2010;98(1):48–56. doi: 10.1016/j.bpj.2009.09.043 20085718 10.1016/j.bpj.2009.09.043 PMC2800967

[16] Mack ET, Cummings L, Perez-Castillejos R. Mathematical model for determining the binding constants between immunoglobulins, bivalent ligands, and monovalent ligands. Anal Bioanal Chem. 2011;399(4):1641–52. doi: 10.1007/s00216-010-4477-y 21161645 10.1007/s00216-010-4477-y

[17] Mack ET, Snyder PW, Perez-Castillejos R, Bilgicer B, Moustakas DT, Butte MJ, et al Dependence of avidity on linker length for a bivalent ligand-bivalent receptor model system. J Am Chem Soc. 2012;134(1):333–45. doi: 10.1021/ja2073033 22088143 10.1021/ja2073033 PMC3272676

[18] Lollar P, Winzor DJ. Reconciliation of classical and reacted-site probability approaches to allowance for ligand multivalence in binding studies. J Mol Recognit. 2014;27(2):73–81. doi: 10.1002/jmr.2335 24436124 10.1002/jmr.2335 PMC3899739

[19] Calvert PD, Nichol LW, Sawyer WH. Binding equations for interacting systems comprising multivalent acceptor and bivalent ligand: application to antigen-antibody systems. J Theor Biol. 1979;80(2):233–47. 529802 10.1016/0022-5193(79)90208-x

[20] Hubble J. A model of multivalent ligand-receptor equilibria which explains the effect of multivalent binding inhibitors. Mol Immunol. 1999;36(1):13–8. 10369416 10.1016/s0161-5890(98)00116-3

[21] Diestler DJ, Knapp EW. Statistical thermodynamics of the stability of multivalent ligand-receptor complexes. Phys Rev Lett. 2008;100(17):178101 doi: 10.1103/PhysRevLett.100.178101 18518340 10.1103/PhysRevLett.100.178101

[22] Diestler DJ, Knapp EW. Statistical Mechanics of the Stability of Multivalent Ligand−Receptor Complexes. The Journal of Physical Chemistry C. 2010;114(12):5287–304.

[23] Kolb HC, Finn MG, Sharpless KB. Click Chemistry: Diverse Chemical Function from a Few Good Reactions. Angew Chem Int Ed Engl. 2001;40(11):2004–21. 11433435 10.1002/1521-3773(20010601)40:11<2004::AID-ANIE2004>3.0.CO;2-5

[24] Debets MF, van Hest JC, Rutjes FP. Bioorthogonal labelling of biomolecules: new functional handles and ligation methods. Org Biomol Chem. 2013;11(38):6439–55. doi: 10.1039/c3ob41329b 23969529 10.1039/c3ob41329b

[25] Patterson DM, Nazarova LA, Prescher JA. Finding the right (bioorthogonal) chemistry. Acs Chem Biol. 2014;9(3):592–605. doi: 10.1021/cb400828a 24437719 10.1021/cb400828a

[26] Barany F, Pingle M, Bergstrom D, Gardina SF, inventors; Cornell University, USA; Purdue Research Foundation. assignee. Coferons and methods of making and using them patent WO2009126290A2. 2009.

[27] Wanner J, Romashko D, Werner DS, May EW, Peng Y, Schulz R, et al Reversible linkage of two distinct small molecule inhibitors of Myc generates a dimeric inhibitor with improved potency that is active in myc over-expressing cancer cell lines. PLoS One. 2015;10(4):e0121793 doi: 10.1371/journal.pone.0121793 25875098 10.1371/journal.pone.0121793 PMC4398458

[28] Sommerhoff CP, Bode W, Pereira PJB, Stubbs MT, Stürzebecher J, Piechottka GP, et al The structure of the human βII-tryptase tetramer: Fo(u)r better or worse. Proceedings of the National Academy of Sciences. 1999;96(20):10984–91. 10.1073/pnas.96.20.10984 PMC34230 10500112

[29] Vollmuth F, Blankenfeldt W, Geyer M. Structures of the dual bromodomains of the P-TEFb-activating protein Brd4 at atomic resolution. J Biol Chem. 2009;284(52):36547–56. doi: 10.1074/jbc.M109.033712 19828451 10.1074/jbc.M109.033712 PMC2794770

[30] Kerns RJ, Rybak MJ, Kaatz GW, Vaka F, Cha R, Grucz RG, et al Structural features of piperazinyl-linked ciprofloxacin dimers required for activity against drug-resistant strains of Staphylococcus aureus. Bioorg Med Chem Lett. 2003;13(13):2109–12. 12798315 10.1016/s0960-894x(03)00376-7

[31] Kramer RH, Karpen JW. Spanning binding sites on allosteric proteins with polymer-linked ligand dimers. Nature. 1998;395(6703):710–3. doi: 10.1038/27227 9790193 10.1038/27227

[32] Busnelli M, Kleinau G, Muttenthaler M, Stoev S, Manning M, Bibic L, et al Design and Characterization of Superpotent Bivalent Ligands Targeting Oxytocin Receptor Dimers via a Channel-Like Structure. J Med Chem. 2016;59(15):7152–66. doi: 10.1021/acs.jmedchem.6b00564 27420737 10.1021/acs.jmedchem.6b00564

[33] Andersen J, Ladefoged LK, Kristensen TN, Munro L, Grouleff J, Stuhr-Hansen N, et al Interrogating the Molecular Basis for Substrate Recognition in Serotonin and Dopamine Transporters with High-Affinity Substrate-Based Bivalent Ligands. ACS Chem Neurosci. 2016;7(10):1406–17. doi: 10.1021/acschemneuro.6b00164 27425420 10.1021/acschemneuro.6b00164

[34] Saiz L, Vilar JM. Stochastic dynamics of macromolecular-assembly networks. Mol Syst Biol. 2006;2:2006 0024. 10.1038/msb4100061 PMC1681493 16738569

[35] Vilar JM, Saiz L. CplexA: a Mathematica package to study macromolecular-assembly control of gene expression. Bioinformatics. 2010;26(16):2060–1. doi: 10.1093/bioinformatics/btq328 20562419 10.1093/bioinformatics/btq328

[36] Jencks WP. On the attribution and additivity of binding energies. Proc Natl Acad Sci U S A. 1981;78(7):4046–50. 16593049 10.1073/pnas.78.7.4046 PMC319722

[37] Zhou HX, Gilson MK. Theory of free energy and entropy in noncovalent binding. Chem Rev. 2009;109(9):4092–107. doi: 10.1021/cr800551w 19588959 10.1021/cr800551w PMC3329805

[38] Saiz L, Vilar JM. DNA looping: the consequences and its control. Curr Opin Struct Biol. 2006;16(3):344–50. doi: 10.1016/j.sbi.2006.05.008 16714105 10.1016/j.sbi.2006.05.008

[39] Saiz L, Vilar JM. Multilevel deconstruction of the In vivo behavior of looped DNA-protein complexes. PLoS One. 2007;2(4):e355 doi: 10.1371/journal.pone.0000355 17406679 10.1371/journal.pone.0000355 PMC1831498

[40] Cang S, Iragavarapu C, Savooji J, Song Y, Liu D. ABT-199 (venetoclax) and BCL-2 inhibitors in clinical development. J Hematol Oncol. 2015;8:129 doi: 10.1186/s13045-015-0224-3 26589495 10.1186/s13045-015-0224-3 PMC4654800

[41] Vilar JM, Saiz L. Control of gene expression by modulated self-assembly. Nucleic Acids Res. 2011;39(16):6854–63. doi: 10.1093/nar/gkr272 21602261 10.1093/nar/gkr272 PMC3167614

[42] Vilar JM, Saiz L. Reliable prediction of complex phenotypes from a modular design in free energy space: an extensive exploration of the lac operon. ACS Synth Biol. 2013;2(10):576–86. doi: 10.1021/sb400013w 23654358 10.1021/sb400013w

