# Supplementary material for: A general model for predicting the binding affinity of reversibly and irreversibly dimerized ligands
Source: PLoS One. 2017 Nov 22;12(11):e0188134. doi: 10.1371/journal.pone.0188134 (PMC5699851; doi:10.1371/journal.pone.0188134)
Supplement: S1 File — (PDF) [file pone.0188134.s001.pdf]

Supporting Information

for

A general model for predicting the binding affinity of reversibly and irreversibly dimerized ligands

Kenneth W. Foreman

BlinkBio, Inc., 130 Scripps Way #2B2, Jupiter, FL 33458

Derivation of equation 20:

Since R is the total fraction of target sites occupied, the following relation must hold,

$$2T_0R = T_0(F_{1u} + 2F_{22} + 2F_{21}). \quad \text{(S1)}$$

Cancelling the factors of T_0 in S1 and substituting the definitions from equations 18 and 19 yields

$$2R = (2F_T[1 - F_T][1 - F_{21}] + 2F_T^2[1 - F_{21}] + 2F_{21}). \quad \text{(S2)}$$

Cancelling the factor of 2 and simplifying the right hand side of equation S2 yields equation 20.

Derivation of equation 23:

Analogous to equation 20, we may define the fraction R' of ligand sites which are occupied as

$$R' = F_{L,Dim} + (1 - F_{L,Dim})F_L, \quad \text{(S3)}$$

where $F_{L,Dim}$ is the fraction of bivalents in the 1:1 state and F_L is the fraction of monomers from the bivalent ligands bound to target, excluding the 1:1 state. R' and R, as well as $F_{L,Dim}$ and F_{21} are related by mass balance equations identical to equation 3. Specifically, $T_0R=L_0R'$ and $T_0F_{21}=L_0F_{L,Dim}$. Substituting these into equation S3 and solving for F_L yields equation 23.

Derivation of equation 25:

Using the definition of R from equation 20,

$$1 - R = 1 - F_{21} - (1 - F_{21})F_T. \quad \text{(S4)}$$

Factoring the right hand side of equation S4 produces

$$1 - R = (1 - F_{21})(1 - F_T). \quad \text{(S5)}$$

Substituting equation S5 into equation 24 yields

$$2T_0F_T(1 - R) = 2T_0R(1 - R)(1 - F_{T,Dim}). \quad \text{(S6)}$$

Cancelling equivalent terms in equation S6 gives equation 25.

Derivation of equation 27:

The definition of molarity is moles of a substance per L. The one free monomer from the bivalent ligand is ~1.66×10^{-24} moles. The volume of the sampled sphere is

$$\frac{4\pi R_p^3}{3}, \tag{S7}$$

with R_p in angstroms. With 10^{10} angstroms in every meter, 10^6 cm^3 in every m^3, and 10^3 cm^3 in every L, S7 becomes approximately $4.18879\times10^{-27}R_p^3$. Taking the ratio produces the right hand side of the defining equation 27.

Derivation of equation 28:

Starting from equation 4 and rearranging to take a standard quadratic form produces

$$F_{T,Dim}^2 T_{0,eff} - F_{T,Dim}\left(T_{0,eff} + L_{0,eff} + K_D\right) + L_{0,eff} = 0. \tag{S8}$$

Recognizing that $T_{0,eff}=L_{0,eff}$ in this treatment and dividing through by $L_{0,eff}$ gives

$$F_{T,Dim}^2 - F_{T,Dim}\left(2 + K_D/L_{0,eff}\right) + 1 = 0. \tag{S9}$$

Putting the solution into standard form and simplifying gives equation 28.

Derivation of the balance point between 1:1 and 1:2 complexes:

From equation 28, when $F_{T,Dim}=½$,

$$F_{T,Dim} = 1/2 = 1 - K_D/2L_{0,eff}\left(\sqrt{1 + 4L_{0,eff}/K_D} - 1\right). \tag{S10}$$

Rearranging so that the square root is on one side, we get

$$\frac{L_{0,eff}}{K_D} + 1 = \sqrt{1 + 4L_{0,eff}/K_D}. \tag{S11}$$

Squaring and consolidating terms yields

$$\left(\frac{L_{0,eff}}{K_D}\right)^2 - 2L_{0,eff}/K_D = 0. \tag{S12}$$

The two roots are $L_{0,eff}=0$ and $2K_D$. The zero is a false root.

Derivation of equation 31:

Case 1: Unbound target binding unbound ligand

$$K_D = \frac{T_0(1-F_T)^2(1-F_{21})L_0(1-F_L)^2(1-F_{L,Dim})}{2T_0F_T(1-F_T)(1-F_{21})(1-F_L)/4} = \frac{2(1-F_T)L_0(1-F_L)(1-F_{L,Dim})}{F_T}. \tag{S13}$$

Case 2: Unbound target binding singly bound ligand

$$K_D = \frac{T_0(1-F_T)^2(1-F_{21})2L_0F_L(1-F_L)\left(1-F_{L,Dim}\right)/2}{2T_0F_T(1-F_T)(1-F_{21})F_L/4} = \frac{2(1-F_T)L_0(1-F_L)\left(1-F_{L,Dim}\right)}{F_T}. \quad \text{(S14)}$$

Case 3: Target singly bound with singly bound ligand binding unbound ligand

$$K_D = \frac{2T_0F_T(1-F_T)(1-F_{21})(1-F_L)L_0(1-F_L)^2\left(1-F_{L,Dim}\right)/4}{T_0F_T^2(1-F_{21})(1-F_L)^2/4} = \frac{2(1-F_T)L_0(1-F_L)\left(1-F_{L,Dim}\right)}{F_T}. \quad \text{(S15)}$$

Case 4: Target singly bound with singly bound ligand binding singly bound ligand

$$K_D = \frac{2T_0F_T(1-F_T)(1-F_{21})(1-F_L)2L_0F_L(1-F_L)\left(1-F_{L,Dim}\right)/8}{T_0F_T^2(1-F_{21})F_L(1-F_L)/4} = \frac{2(1-F_T)L_0(1-F_L)\left(1-F_{L,Dim}\right)}{F_T}. \quad \text{(S16)}$$

Case 5: Target singly bound with doubly bound ligand binding unbound ligand

$$K_D = \frac{2T_0F_T(1-F_T)(1-F_{21})F_LL_0(1-F_L)^2\left(1-F_{L,Dim}\right)/4}{T_0F_T^2(1-F_{21})F_L(1-F_L)/4} = \frac{2(1-F_T)L_0(1-F_L)\left(1-F_{L,Dim}\right)}{F_T}. \quad \text{(S17)}$$

Case 4: Target singly bound with doubly bound ligand binding singly bound ligand

$$K_D = \frac{2T_0F_T(1-F_T)(1-F_{21})F_L2L_0F_L(1-F_L)\left(1-F_{L,Dim}\right)/8}{T_0F_T^2(1-F_{21})F_L^2/4} = \frac{2(1-F_T)L_0(1-F_L)\left(1-F_{L,Dim}\right)}{F_T}. \quad \text{(S18)}$$

Defining R' as the fraction of ligand sites bound and noting the parallel between two ligand sites and two target sites, we coopt equation S5 to read

$$1 - R' = \left(1 - F_{L,Dim}\right)(1 - F_L). \quad \text{(S19)}$$

Since total sites bound from both sides must be equal,

$$R' = \frac{T_0}{L_0}R. \quad \text{(S20)}$$

Substituting equations S30 and S29 into any of equations S25-S28 gives

$$K_D = \frac{2(1-F_T)L_0\left(1-\frac{T_0}{L_0}R\right)}{F_T} = 2\,\frac{(1-F_T)}{F_T}(L_0 - T_0R). \quad \text{(S21)}$$

Derivation of equation 35:

By definition, K_2 is

$$K_2 = \frac{[T_0(1-F_T)^2(1-F_{21})]\left[L_0(1-F_L)^2\left(1-F_{L,Dim}\right)\right]}{F_{21}T_0/2}, \quad \text{(S22)}$$

which when rearranged gives

$$K_2 = 2L_0\frac{[(1-F_T)^2(1-F_{21})]\left[(1-F_L)^2\left(1-F_{L,Dim}\right)\right]}{F_{21}} = 2L_0\frac{(1-F_T)(1-R)(1-F_L)^2\left(1-F_{L,Dim}\right)}{F_{21}}, \quad \text{(S23)}$$

where the second equality comes from substitution of equation S5. Since $L_0 >> T_0$, equation S23 may be approximated as

$$K_2 \approx 2L_0 \frac{(1-F_T)(1-R)}{F_{21}}. \quad \text{(S24)}$$

Substituting equations 25 and 26 into S24 yields

$$K_2 = 2L_0 \frac{\left[1-R\left(1-F_{T,Dim}\right)\right](1-R)}{\frac{R}{1-R\left(1-F_{T,Dim}\right)}} = 2L_0 \frac{\left[1-R\left(1-F_{T,Dim}\right)\right]^2}{F_{T,Dim}} \frac{1-R}{R}. \quad \text{(S25)}$$

At R=½, equation S25 becomes

$$K_2 = L_0 \frac{\left[\left(1+F_{T,Dim}\right)\right]^2}{2F_{T,Dim}}. \quad \text{(S26)}$$

Substitution of equation 32 into equation S26 produces equation 35.

Derivation of equation 39:

From equation 38, cancel terms and place all but square-root terms on the right hand side of the equation to get

$$\sqrt{1+4L_{0,eff}/K_D} = 1 + 4L_{0,eff}. \quad \text{(S27)}$$

Squaring both sides and placing in quadratic form yields

$$0 = 4L_{0,eff}(2 - 1/K_D) + 16L_{0,eff}^2. \quad \text{(S28)}$$

The roots are 0 (disallowed) and $1/(4K_D)-1/2 \approx 1/(4K_D)$ [since $K_D<<1$].

Derivation of equation 40:

Taking the limit of equation 28 as $L_{0,eff}$ becomes much greater than K_D yields

$$F_{T,Dim} = 1 - \sqrt{K_D/L_{0,eff}}. \quad \text{(S29)}$$

Substituting S29 into equation 35 produces

$$K_2 = \frac{K_D}{4}\left(\frac{1}{F_{T,Dim}} - F_{T,Dim}\right) = \frac{K_D}{4}\left(\frac{1}{1-\sqrt{\frac{K_D}{L_{0,eff}}}} - 1 + \sqrt{\frac{K_D}{L_{0,eff}}}\right) = \frac{K_D}{4}\left(1 + \frac{\sqrt{\frac{K_D}{L_{0,eff}}}}{1-\sqrt{\frac{K_D}{L_{0,eff}}}} - 1 + \sqrt{\frac{K_D}{L_{0,eff}}}\right).$$

(S30)

Simplifying equation S30 gives

$$K_2 = \frac{K_D\sqrt{\frac{K_D}{L_{0,eff}}}}{4}\left(\frac{1}{1-\sqrt{\frac{K_D}{L_{0,eff}}}} + 1\right) \approx \frac{K_D\sqrt{\frac{K_D}{L_{0,eff}}}}{4}(2) = \frac{K_D\sqrt{\frac{K_D}{L_{0,eff}}}}{2}. \quad \text{(S31)}$$

The right hand side of equation S31 is equivalent to the right hand side of equation 40.

Derivation of equation 52:

After cancellation of equivalent terms on the left and right hand sides of equation 51, one gets

$$\frac{L_u}{L_1} = \frac{D_u}{(1/2)D_1}. \quad \text{(S32)}$$

Substitution of equations 46-49 into S32 gives

$$\frac{(1-F_{L,M})F_{ML}}{F_{L,M}F_{ML}} = \frac{(1-F_{L,D})^2 F_{DL}}{F_{L,D}(1-F_{L,D})F_{DL}}, \quad \text{(S33)}$$

or equivalently,

$$\frac{(1-F_{L,M})}{F_{L,M}} = \frac{(1-F_{L,D})}{F_{L,D}}. \quad \text{(S34)}$$

Solving equation S34 for either $F_{L,M}$ or $F_{L,D}$ yields the same result as equation 52.

Derivation of equation 53:

Since R' is the fraction of all monomers from bivalent ligands bound to target,

$$L_0R' = L_0(L_1 + D_1/2 + D_{22} + D_{21}). \quad \text{(S35)}$$

Substituting equations 47, 49, 50, and 52 into S35 generates

$$R' = F_L F_{ML} + F_L(1 - F_L)F_{DL} + F_L^2 F_{DL} + D_{21}. \quad \text{(S36)}$$

Simplifying S36 and substituting from equation 42 produces equation 53.

Derivation of equation 54:

Starting with the immediate equality on the right hand side of equation 53 and solving for F_L yields

$$\frac{R\frac{2T_0}{L_0} - D_{21}}{1-D_{21}} = F_L. \quad \text{(S37)}$$

The amount of ligand bound to the target in the 1:1 state must equal the number of target sites occupied in that state, which is stated mathematically as $L_0D_{21}=2T_0F_{21}$, which when substituted into S37 and simplifying gives

$$F_L = \frac{R\frac{2T_0}{L_0} - 2\frac{T_0F_{21}}{L_0}}{1-2\frac{T_0F_{21}}{L_0}} = \frac{2T_0(R-F_{21})}{L_0-2T_0F_{21}}, \quad \text{(S38)}$$

which is equation 54.

Derivation of equation 55:

For two unbound monomers dimerizing,

$$K_{Dim} = \frac{L_0^2 L_u^2}{1/2 L_0 D_u} = 2L_0 \frac{L_u^2}{D_u}. \qquad \text{(S39)}$$

For one bound monomer and one unbound monomer dimerizing,

$$K_{Dim} = \frac{L_0^2 L_u L_1}{1/4 L_0 D_1} = 4L_0 \frac{L_u L_1}{D_1}. \qquad \text{(S40)}$$

For two bound monomers (not on the same target) dimerizing,

$$K_{Dim} = \frac{L_0^2 L_1^2}{1/2 L_0 D_{22}} = 2L_0 \frac{L_1^2}{D_{22}}. \qquad \text{(S41)}$$

Multiplying through with the denominators in each of equations S39-S41 and adding them together gives

$$K_{Dim}(D_u + D_1 + D_{22}) = 2L_0(L_u^2 + 2L_u L_1 + L_1^2). \qquad \text{(S42)}$$

The left hand side in parentheses is just F_{LD}, while the right hand side in parentheses is F_{LM}^2. Solving for K_{Dim} gives equation 55.

Derivation of equation 56:

Starting from equation 55 and substituting in equation 42 yields

$$K_{Dim} = 2L_0 \frac{(1-F_{DL}-D_{21})^2}{F_{DL}}. \qquad \text{(S43)}$$

Rewriting equation S43 in standard quadratic form for F_{DL} looks like

$$0 = (1 - D_{21})^2 - F_{DL}[2(1 - D_{21}) + K_{Dim}/2L_0] + F_{DL}^2, \qquad \text{(S44)}$$

implying the solutions for F_{DL} are

$$F_{DL} = (1 - D_{21}) + K_{Dim}/4L_0 \pm \sqrt{(1 - D_{21})\, K_{Dim}/2L_0 + K_{Dim}^2/16L_0^2}. \qquad \text{(S45)}$$

As L_0 goes to 0, the only way F_{DL} remains finite for a finite K_{Dim} is if the negative root is selected. Factoring out $K_{Dim}/4L_0$ from the root and simplifying produces equation 56.

Confirmation that dimer limit is obtained when L_0>>K_{Dim}:

Substituting equations 42, 43, 44, 46, 47, and 52 into equation 51 and cancelling terms yields

$$\frac{(1-F_T)L_0(1-F_L)}{F_T/(1-D_{21})} = K_D. \qquad \text{(S46)}$$

Substituting equations 21 and 54 into equation S46 while noting that $L_0D_{21}=2T_0F_{21}$, and simplifying yields

$$\frac{(1-R)(L_0-2T_0R)}{(R-F_{21})} = K_D. \quad \text{(S47)}$$

Substituting R=½ and acknowledging $L_0 >> T_0$ implies,

$$\frac{1}{(1-2F_{21})} L_0 \approx K_D. \quad \text{(S48)}$$

Equation S48 is a general result, true regardless of the relative values of L_0 and K_{Dim}. When $L_0 >> K_{Dim}$, equation 24 is the controlling boundary condition, with the understanding that the calculation of $F_{T,Dim}$ uses a value of R_p which is twice the maximum linker length of that of the monomeric ligand. But the definition of F_T has not changed, so equations 21 and 25 still hold and thus, equation 32 is the final result; that is

$$K_D = \frac{1+F_{T,Dim}}{1-F_{T,Dim}} L_0. \quad \text{(S49)}$$

Note that the factor of 2 in equation 32 is absorbed into the L_0 term when describing the current system since the concentration of dimer is half that of the monomer.

Derivation of equation 58:

Substituting equations 45, 47, and 56 into equation 57 and cancelling terms yields

$$F_T^2(1-F_{21})\left(\frac{F_{ML}}{1-D_{21}}\right)^2 = R^2\left[\frac{K_{Dim}}{4L_0}\left(\sqrt{1+\frac{8L_0}{K_{Dim}}}-1\right)\right]^2\left(1-F_{L,Dim}\right). \quad \text{(S50)}$$

Substituting equations 21, 42, and 56 reduces equation S50 to

$$\frac{(R-F_{21})^2}{(1-F_{21})}\left(\frac{\frac{K_{Dim}}{4L_0}\left(\sqrt{1+\frac{8L_0}{K_{Dim}}(1-D_{21})}-1\right)}{1-D_{21}}\right)^2 = R^2\left[\frac{K_{Dim}}{2L_0}\left(\sqrt{1+\frac{4L_0}{K_{Dim}}}-1\right)\right]^2\left(1-F_{L,Dim}\right). \quad \text{(S51)}$$

Since $L_0D_{21}=2T_0F_{21}$, in the limit of $L_0 >> T_0$, $D_{21} << 1$, permitting equation S51 to be rewritten as

$$(R-F_{21})^2 = R^2\left(1-F_{L,Dim}\right)(1-F_{21}). \quad \text{(S52)}$$

Equation S52 can be recast as a quadratic in F_{21},

$$F_{21}^2 - F_{21}R\left[2-R\left(1-F_{L,Dim}\right)\right] + R^2F_{L,Dim} = 0, \quad \text{(S53)}$$

whose physically relevant root (taken by observing the desired behavior at R=1) is

$$F_{21} = R - R^2\frac{\left(1-F_{L,Dim}\right)}{2} - R\sqrt{R^2\left[\frac{\left(1-F_{L,Dim}\right)}{2}\right]^2 + (1-R)\left(1-F_{L,Dim}\right)}, \quad \text{(S54)}$$

which is equation 58.

Derivation of effective concentrations for dimerizing moieties both bound to a single target:

Both moieties are treated as being in spheres of radius $R_p/2$ whose centers are separated by a distance

R_s. The volume of overlap between spheres is given by

$$V_o = \frac{\pi}{4}\left[\left(R_p - \frac{\sqrt{R_p^2 - R_s^2}}{3}\right)\left(R_p^2 - R_s^2\right)\right], \tag{S55}$$

while the volume of each sphere is

$$V_{sph} = \frac{\pi R_p^3}{6}. \tag{S56}$$

The effective concentration is given by the number of moieties per total sampled volume, or

$$L_{0,eff} = \frac{2}{2V_{sph} - V_o} = \frac{1}{V_{sph} - V_o/2} = \frac{1}{V_{sph}}\frac{1}{1 - V_o/2V_{sph}}. \tag{S57}$$

Clearly, when there is no overlap, the far right hand side of equation S57 becomes $1/V_{sph}$, while in the other limit of complete overlap (V_o=V_{sph}), the far right hand side becomes $2/V_{sph}$. Since the denominator is linear in V_o, the multiplicative factor to $1/V_{sph}$ can only vary between 1 and 2.

Derivation of equation 60:

Equation 55 defines the equilibrium for dimerization, where F_{ML} is now 1-F_{DL}, $L_{0,eff}$ replaces L_0, and F_{DL} is $F_{L,Dim}$. Thus equation 55 (or S39) can be written

$$K_{Dim} = 2L_{0,eff}\frac{\left(1 - F_{L,Dim}\right)^2}{F_{L,Dim}}. \tag{S58}$$

Rearranging equation S58 as a quadratic in $F_{L,Dim}$ yields

$$0 = F_{L,Dim}^2 - F_{L,Dim}\left(2 + \frac{K_{Dim}}{2L_{0,eff}}\right) + 1, \tag{S59}$$

which produces the physically meaningful root, equation 60.

Derivation of equation 61:

The fraction of targets with two monomers bound is given by the fraction of targets with two sites occupied, scaled by fraction of monomer at each site, namely, from equation 45,

$$F_{22,mm} = F_T^2(1 - F_{21})\left[\frac{L_1}{L_1 + D_1/2 + D_{22}}\right]^2, \tag{S60}$$

which, in the limit of F_{21}=0, reduces to

$$F_{22,mm} = R^2\left[\frac{F_L F_{ML}}{F_L F_{ML} + F_L(1 - F_L)F_{DL} + F_L^2 F_{DL}}\right]^2 = R^2 {F_{ML}}^2, \tag{S61}$$

Similarly, the fraction of targets with one site containing an otherwise unbound dimer and the other site unoccupied is, from equation 44,

$$F_{1,D1} = 2F_T(1-F_T)(1-F_{21})\frac{D_1/2}{L_1+D_1/2+D_{22}}, \quad \text{(S62)}$$

which, in the limit of $F_{21}=0$, reduces to

$$F_{1,D1} = 2R(1-R)\frac{F_L(1-F_L)F_{DL}}{F_L} \approx 2R(1-R)F_{DL}, \quad \text{(S63)}$$

where the approximation holds since $L_0 >> T_0$. The fractional contributions from equations 26 ($F_{21,e26}$) and 58 ($F_{21,e58}$) can thus be written as

$$F_{21} = \frac{F_{22,mm}}{F_{22,mm}+F_{1,D1}}F_{21,e58} + \frac{F_{1,D1}}{F_{22,mm}+F_{1,D1}}F_{21,e26}. \quad \text{(S64)}$$

Substitution of equations 26, 58, S61, and S63 into S64 yields equation 61, noting that a factor of R can be cancelled between numerator and denominator in the fractions.

Derivation of equation 63:

Equation S48 is the appropriate expression for K_D, and substitution of F_{21} is required. In order to simplify the readability, we evaluate the denominator on the right hand side of equation S48 first and then substitute afterward. Setting R=½ in equation 61 and substituting equation 42 gives

$$F_{21} = \frac{(1-F_{DL})^2}{F_{DL}^2+1}\frac{1}{2}\left[1 - \frac{1-F_{L,Dim}}{4} - \sqrt{\left[\frac{1-F_{L,Dim}}{4}\right]^2 + \frac{1-F_{L,Dim}}{2}}\right] + \frac{2F_{DL}}{F_{DL}^2+1}\frac{F_{T,Dim}}{1+F_{T,Dim}}. \quad \text{(S65)}$$

Substituting equation S65 into the denominator from equation S48 and simplifying yields

$$1-2F_{21} = \frac{(1-F_{DL})^2}{F_{DL}^2+1}\left[\frac{1-F_{L,Dim}}{4} + \sqrt{\left[\frac{1-F_{L,Dim}}{4}\right]^2 + \frac{1-F_{L,Dim}}{2}}\right] + \frac{2F_{DL}}{F_{DL}^2+1}\left[\frac{1-F_{T,Dim}}{1+F_{T,Dim}}\right]. \quad \text{(S66)}$$

From the definition of F_{DL} in equation 56 and the understanding that $D_{21}<<1$ since $L_0>>T_0$, one gets

$$1-F_{DL} \approx \frac{K_{Dim}}{4L_0}\left(\sqrt{1+\frac{8L_0}{K_{Dim}}}-1\right), \quad \text{(S67)}$$

and from the definition of $F_{L,Dim}$ and $L_{0,eff}$ in equations 60 and 59, respectively, one gets

$$1-F_{L,Dim} = \frac{K_{Dim}R_p^3}{6342.4}\left(\sqrt{1+\frac{12684.8}{K_{Dim}R_p^3}}-1\right), \quad \text{(S68)}$$

while the definition of $F_{T,Dim}$ from equations 28 and 30 gives

$$1-F_{T,Dim} = \frac{K_D R_p^3}{792.8}\left(\sqrt{1+\frac{1585.6}{K_D R_p^3}}-1\right). \quad \text{(S69)}$$

Substituting equations S67-S69 into equation S66 yields

$$1-2F_{21}=\left\{\left[\frac{K_{Dim}}{4L_0}\left(\sqrt{1+\frac{8L_0}{K_{Dim}}}-1\right)\right]^2\left\{\frac{\frac{K_{Dim}R_p^3}{6342.4}\left(\sqrt{1+\frac{12684.8}{K_{Dim}R_p^3}}-1\right)}{4}+\sqrt{\left[\frac{\frac{K_{Dim}R_p^3}{6342.4}\left(\sqrt{1+\frac{12684.8}{K_{Dim}R_p^3}}-1\right)}{4}\right]^2+\frac{\frac{K_{Dim}R_p^3}{6342.4}\left(\sqrt{1+\frac{12684.8}{K_{Dim}R_p^3}}-1\right)}{2}}\right\}\right.$$
$$\left.+2\left[1-\frac{K_{Dim}}{4L_0}\left(\sqrt{1+\frac{8L_0}{K_{Dim}}}-1\right)\right]\left[\frac{\frac{K_DR_p^3}{792.8}\left(\sqrt{1+\frac{1585.6}{K_DR_p^3}}-1\right)}{2-\frac{K_DR_p^3}{792.8}\left(\sqrt{1+\frac{1585.6}{K_DR_p^3}}-1\right)}\right]\right\}\Bigg/\left\{1+\left[1-\frac{K_{Dim}}{4L_0}\left(\sqrt{1+\frac{8L_0}{K_{Dim}}}-1\right)\right]^2\right\}$$

. (S70)

Equation S70 as substituted into equation S48 is the one used in all of the calculations. For a given value of K_{Dim}, R_p, and K_D, L_0 is solved for numerically.
